# “It’s hard for us men to go to the clinic. We naturally have a fear of hospitals.” Men’s risk perceptions, experiences and program preferences for PrEP: A mixed methods study in Eswatini

**DOI:** 10.1371/journal.pone.0237427

**Published:** 2020-09-23

**Authors:** Astrid Berner-Rodoreda, Pascal Geldsetzer, Kate Bärnighausen, Anita Hettema, Till Bärnighausen, Sindy Matse, Shannon A. McMahon

**Affiliations:** 1 Institute of Global Health, Ruprecht-Karls-Universität, Heidelberg, Germany; 2 Division of Primary Care and Population Health, Department of Medicine, Stanford University, Stanford, California, United States of America; 3 University of the Witwatersrand School of Public Health, Johannesburg, South Africa; 4 Clinton Health Access Initiative Swaziland, Mbabane, Eswatini; 5 Harvard T.H. Chan School of Public Health, Boston, Massachusetts, United States of America; 6 Eswatini Ministry of Health, Mbabane, Eswatini; 7 Department of International Health, Johns Hopkins Bloomberg School of Public Health, Baltimore, MD, United States of America; George Washington University, UNITED STATES

## Abstract

Few studies on HIV Pre-Exposure Prophylaxis (PrEP) have focused on men who have sex with women. We present findings from a mixed-methods study in Eswatini, the country with the highest HIV prevalence in the world (27%). Our findings are based on risk assessments, in-depth interviews and focus-group discussions which describe men’s motivations for taking up or declining PrEP. Quantitatively, men self-reported starting PrEP because they had multiple or sero-discordant partners or did not know the partner’s HIV-status. Men’s self-perception of risk was echoed in the qualitative data, which revealed that the hope of facilitated sexual performance or relations, a preference for pills over condoms and the desire to protect themselves and others also played a role for men to initiate PrEP. Trust and mistrust and being able or unable to speak about PrEP with partner(s) were further considerations for initiating or declining PrEP. Once on PrEP, men’s sexual behavior varied in terms of number of partners and condom use. Men viewed daily pill-taking as an obstacle to starting PrEP. Side-effects were a major reason for men to discontinue PrEP. Men also worried that taking anti-retroviral drugs daily might leave them mistaken for a person living with HIV, and viewed clinic-based PrEP education and initiation processes as a further obstacle. Given that men comprise only 29% of all PrEP users in Eswatini, barriers to men’s uptake of PrEP will need to be addressed, in terms of more male-friendly services as well as trialing community-based PrEP education and service delivery.

## Introduction

Daily pre-exposure prophylaxis (PrEP) with antiretroviral drugs has been recommended by the World Health Organization (WHO) as an additional prevention tool since 2012 [1]. Trials with daily TDF or TDF/FTC (alternatively TDF/3TC) have shown that PrEP is efficacious in reducing the risk of contracting HIV by around 90% when taken consistently [2]. The WHO gradually extended recommendations for PrEP use from men who have sex with men (MSM), transgender women and sero-discordant couples to all HIV-negative people at substantial risk of an HIV infection, defined as HIV incidence greater than 3 per 100 person–years [3].

Not all population groups have, however, benefitted from PrEP to the same extent. While many countries continue to focus on MSM, female and male sex workers and transgender women and men, a number of predominantly African countries have extended or are extending PrEP to the wider population, see www.prepwatch.org/resource/global-prep-tracker, yet the PrEP focus in Sub-Saharan Africa is on women rather than men [4] and echoes a general gender trend in HIV interventions in Africa [5,6]. Despite the fact that men who have sex with women (MSW) have been lagging behind in the use of HIV testing services [3,6], presented late with HIV [7–9] and experienced worse health outcomes and higher rates of HIV-related deaths than women [10–18], they are rarely seen as an HIV risk group in their own right [6,19,20].

PrEP could offer a new HIV prevention method for MSW, yet so far very few PrEP studies have been conducted with MSW beyond showing the efficacy of PrEP in preventing new infections in sero-discordant couples [21,22]. In a systematic review by Koechlin and colleagues [23], only two studies relate to MSW and PrEP [24,25]. Both describe men's hypothetical views on taking PrEP, as PrEP was not available at the time of research. Additional PrEP studies involving MSW focused on preferences for particular HIV prevention technologies including PrEP [26–28], exploring views and acceptability of PrEP within communities [29,30], among young heterosexual men [31] and among sero-discordant couples wanting to conceive [32]. Only one study documented men (and women’s) actual experiences with PrEP as part of a PrEP trial, highlighting side-effects, adherence, clinic visits and stigma as barriers to PrEP uptake and retention [33]. Our study therefore contributes to the scarce literature on MSW and PrEP.

The Ministry of Health of the Kingdom of Eswatini (formerly Swaziland) with support from Heidelberg University and the Clinton Health Access Initiative conducted one of the first PrEP general population demonstration projects between August 2017 and January 2019 in six public health clinics. Preliminary data revealed a need to focus on men and to include the perspective of community leaders [34,35]. Our nested mixed-methods study examines men’s response to PrEP in order to i) identify the main characteristics of men at risk of an HIV infection and men who take up PrEP, as well as their risk perceptions, ii) explore MSW’s reasons for taking up or declining PrEP and their experiences with PrEP, and iii) describe men’s experience with facility-based PrEP education and delivery and advance MSW’s recommendations for further PrEP education and service delivery.

## Methods

### Study setting: Eswatini

The Kingdom of Eswatini is a landlocked country in Southern Africa with a population of approximately 1.4 million and the highest HIV prevalence in the world, currently at 27% with prevalence for women at 35% and men at 20.4% [36]. For men, the age group 30-34 years shows the highest HIV incidence (3.1 per 100 person-years) [37], while for women incidence peaks in the age groups 18-24 and 35-39 [37]. Nationally, men in sero-discordant relationships and those with a partner of unknown HIV status showed a 16% and 30% HIV incidence respectively [37].

### PrEP Demonstration study

This 18-month PrEP Demonstration Project targeted at the general population was approved by the Eswatini Ministry of Health National Health Research Review Board (MH/599C/IRB0009688/NHRRB538/17) and the USA Chesapeake Institutional Review Board (Pro00021864) with an exemption granted by the Heidelberg Ethics Commission.

It was conducted in six government clinics in the Hhohho Region in the North-West of Eswatini. Nurses provided information on PrEP each morning as part of their general health talks on HIV and TB issues. Clients were offered PrEP if they tested negative for HIV, consented to undergo a risk assessment (see S1 File), were interested in starting PrEP, and identified to be ‘at risk’ based on having experienced the following risk situations in the last six months: unprotected sex, a partner of unknown or HIV-positive status, a sexually transmitted infection (STI), sex under the influence of alcohol or having made use of post-exposure prophylaxis (PEP). Clients could also be offered PrEP if they belonged to one of the following target populations defined by the Eswatini Government as: women between 16 and 25 years of age, those in a relationship with an HIV-positive partner, sex workers, men having sex with men, those with a current sexually transmitted infection, pregnant women, and lactating women [38] – the target populations for the overall study were thus more women-focused. If clients fulfilled one of the above named risk criteria, had no contra-indications for TDF/FTC or TDF/3TC and expressed an interest in taking up PrEP, they were initiated on PrEP and received a one month’s supply of PrEP pills on the same day according to the Eswatini protocol [38]. Clients identified as not being at risk could still be initiated on PrEP if they so desired.

### Design, sampling and data collection

This mixed methods study is based on a socio-ecological model [39]. We adapted the model to focus on men as individuals, their social relations and their experience of health clinics. The study draws on quantitative and qualitative data in a concurrent study design. In addition to risk assessment forms and PrEP uptake data, we drew on the views and experiences of PrEP among male clients, PrEP providers, stakeholders, community leaders and community members.

After receiving an initial off-site training on PrEP delivery followed by facility-based onsite training over two days in 2017, nurses in the six PrEP demonstration clinics conducted risk assessments from August 2017 to January 2019 based on the national PrEP implementation framework [38]. Our sample is composed of those undergoing risk assessments and taking up PrEP in all six health clinics. Eligible and interested clients were given a one-month PrEP supply for starting PrEP and were scheduled for follow-up visits after one month, two months and then in three-month intervals. PrEP files and registers were used to document client data.

In September 2017, local research assistants with fluency in siSwati and English (n=5) were trained for five days on qualitative data collection, interview techniques and on recording field notes throughout data collection. Interview guides were piloted and revised as necessary. Qualitative data on men’s perceptions were collected between September 2017 and February 2019 via in-depth interviews (IDIs) and focus group discussions (FGDs). Interviewees and participants in FGDs were purposively selected: clients by PrEP service providers; PrEP service providers upon availability; stakeholders by identifying those who work in HIV prevention in Eswatini; community leaders through the community leadership system and FGD participants by community leaders. Qualitative data collection was based on principles of qualitative research [40,41] and grounded theory [42,43]. The research team conducted FGDs lasting 1-2 hours to gain a broad perspective on HIV and PrEP from various groups such as mobile men (bus-drivers), youth and community leaders (see S2 File). IDIs lasting approximately 30 minutes to one hour were conducted in a private room or space at the respective clinic or organization. The interview guide included open and closed-ended questions about perceptions of daily PrEP as a prevention method (see S3–S11 Files). For those directly engaged with PrEP, experiences and issues related to sexual behavior before and after taking PrEP were also included. Interviews with community leaders included questions on PrEP education and service delivery for men (see S12 and S13 Files). The minimum age for participation was 16 years. Written informed consent was obtained from all participants prior to interviews and FGDs and after they had been informed about the study orally and in writing.

While the study was not designed with an intention of exclusively examining challenges and experiences of PrEP initiation by men, challenges unique to men emerged in early interviews and were probed more pointedly as interviews progressed; the team explored the perspective of MSW towards PrEP by focusing on men’s responses in FGDs and IDIs. IDIs were conducted with men directly engaged with PrEP as well as male PrEP service providers, stakeholders and community leaders, see Table 1.

**Table 1:** Qualitative methods of data collection.

**Focus Group Discussions (FGD)**	**N**
Bus drivers^a^	1
Community Advisory Board members^b^	2
Youth^b^	1
**Total FGDs**	**4**
**In-Depth Interviews (IDI)**	**N**
**Men directly engaged with PrEP**	**34**
PrEP initiators (men who started PrEP)	8
PrEP continuers (men who continued PrEP over some weeks or months)	14
PrEP decliners (men who went through the risk assessment process and declined PrEP)	5
PrEP discontinuers (men who took PrEP for some time and abandoned it)	5
PrEP re-initiators (men who started PrEP, paused, and re-started PrEP)	1
PrEP deferrers (men who had to delay PrEP due to health issues)	1
**Male PrEP providers** (health care workers)	**10**
**Male stakeholders** (public health implementers, national and international NGOs and networks)	**7**
**Male community leaders** (chiefs, village heads, headmen, members of local committees)	**14**
**Total IDIs with men**	**65**

### Data analysis

In order to determine men’s risks, we conducted univariable and multivariable regressions. We regressed whether a client was identified as being at risk of acquiring HIV (conditional on having undergone a risk assessment) onto sex in univariable regression. In multivariable regressions we used the variables sex, ten-year age group, education, relationship status, and whether the participant belonged to at least one target population as stipulated by the Eswatini Government [38] (for details, see PrEP Demonstration Study above). We ran the same regression models for the outcome of taking up PrEP conditional on having been identified as being at risk. These regressions were Poisson regressions with a random intercept for each healthcare facility. Standard errors were additionally adjusted for clustering at the level of the healthcare facility.

All in-depth interviews were audio-recorded. Researchers discussed the data and findings throughout data collection in debriefing sessions [44]. Interviews were transcribed and simultaneously translated into English. Drawing on Grounded Theory [42,43], codes were primarily assigned inductively derived from the responses, yet deductive coding was also used drawn from sections of the interview schedule. As new codes were added to the code-book, interviews were re-coded. Two authors coded the data independently using Nvivo 12 Pro and 11 respectively. Comparing the coding of two interviews showed reliability in the coding. Salient themes from debriefs and coding emerged around men’s risk perception and experience with PrEP as individuals, in relationships and with regard to health facilities and were discussed on a weekly basis between the first and last author. We analyzed quantitative data on men’s risk and uptake of PrEP and qualitative data on men’s perceptions and experiences of PrEP with a view to informing future PrEP population roll-out for MSW uptake.

## Results

### Risk and risk perception

Examining the characteristics of the male sample screened for risk, 44.9% of men were 26-35 years-old, 58.4% had completed secondary schooling and 38.8% had a partner but were not living together, see Table 2. For men initiating PrEP, characteristics were similar to the overall sample except for relationship status. Male PrEP initiators predominantly had multiple partners (41%) and/or lived together with a partner (31.2%).

**Table 2:** Sample characteristics of men and women^‡^.

	Total under-going risk assessment	Men	Women	Total initiating PrEP	Men	Women
N	**2154**	504	1650	**517**	144	373
**At risk**	**1543 (71.2)**	392 (77.8)	1139 (69.0)			
Missing, n (%)	**32 (1.5)**	9 (1.8)	23 (1.4)			
Age, mean (SD^§^)	**29.0 (10.4)**	31.5 (11.4)	28.1 (9.8)	**30.08 (9.13)**	32.04 (10.63)	29.23 (8.38)
**Age group**, n (%)						
16-25 years	**860 (39.9)**	146 (29.0)	714 (43.3)	**167 (32.3)**	38 (26.4)	129 (34.6)
26-35 years	**867 (40.3)**	225 (44.6)	642 (38.9)	**223 (43.1)**	63 (43.8)	160 (42.9)
36-45 years	**282 (13.1)**	80 (15.9)	202 (12.2)	**91 (17.6)**	27 (18.8)	64 (17.2)
>45 years	**115 (5.3)**	50 (9.9)	65 (3.9)	**36 (7.0)**	16 (11.1)	20 (5.4)
Missing, n (%)	**30 (1.4)**	3 (0.6)	27 (1.6)			
**Education**, n (%)						
No formal schooling	**69 (3.2)**	25 (5.0)	44 (2.7)	**28 (5.5)**	12 (8.3)	16 (4.3)
Some or completed primary school	**394 (18.3)**	98 (19.4)	296 (17.9)	**135 (26.5)**	42 (29.2)	93 (24.9)
Some or completed secondary school	**1194 (55.4)**	232 (46.0)	962 (58.3)	**311 (60.02)**	74 (51.4)	237 (63.5)
Some or completed tertiary education	**109 (5.1)**	42 (8.3)	67 (4.1)	**35 (6.8)**	15 (10.4)	20 (5.4)
Missing, n (%)	**388 (18.0)**	107 (21.2)	281 (17.0)	**8 (1.5)**	1 (0.7)	7 (1.9)
**Relationship status**, n (%)						
Multiple partners	**135 (6.3)**	115 (22.8)	20 (1.2)	**68 (13.2)**	59 (41.0)	9 (2.4)
One partner, living together	**687 (31.9)**	104 (20.6)	583 (35.3)	**205 (39.7)**	45 (31.2)	160 (42.9)
One partner, not living together	**918 (42.6)**	155 (30.8)	763 (46.2)	**239 (46.2)**	37 (25.7)	202 (54.2)
Single, no relationship	**63 (2.9)**	26 (5.2)	37 (2.2)	**5 (1.0)**	3 (2.1)	2 (0.5)
Missing, n (%)	**351 (16.3)**	104 (20.6)	247 (15.0)			
Member of a target population**, n (%)	**1183 (54.9)**	148 (29.4)	1035 (62.7)	356 (68.9)	72 (50.0)	284 (76.1)
**Risk Groups**						
***Unprotected sex***						
Missing or Blank	**384 (17.8)**	116 (23.0)	268 (16.2)	5 (1.0)	2 (1.4)	3 (0.8)
*No*	**413 (19.2)**	102 (20.2)	311 (18.8)	84 (16.2)	26 (18.1)	58 (15.5)
*Yes*	**1357 (63.0)**	286 (56.7)	1071 (64.9)	428 (82.8)	116 (80.6)	312 (83.6)
***Partner HIV-positive or unknown HIV-status***						
Missing or Blank	**384 (17.8)**	116 (23.0)	268 (16.2)	5 (1.0)	2 (1.4)	3 (0.8)
*No*	**782 (36.3)**	141 (28.0)	641 (38.8)	107 (20.7)	26 (18.1)	81 (21.7)
*Yes*	**988 (45.9)**	247 (49.0)	741 (44.9)	405 (78.3)	116 (80.6)	289 (77.5)
***Having (had) STIs***						
Missing or Blank	**385 (17.9)**	117 (23.2)	268 (16.2)	6 (1.2)	2 (1.4)	4 (1.1)
*No*	**1548 (71.9)**	280 (55.6)	1268 (76.8)	420 (81.2)	102 (70.8)	318 (85.3)
*Yes*	**221 (10.3)**	107 (21.2)	114 (6.9)	91 (17.6)	40 (27.8)	51 (13.7)
***Use of Post-Exposure Prophylaxis (PEP)***						
Missing or Blank	**386 (17.9)**	119 (23.6)	267 (16.2)	6 (1.2)	3 (2.1)	3 (0.8)
*No*	**1757 (81.6)**	383 (76.0)	1374 (83.3)	505 (97.7)	140 (97.2)	365 (97.9)
*Yes*	**11 (0.5)**	2 (0.4)	9 (0.5)	6 (1.2)	1 (0.7)	5 (1.3)
***Sex under influence of drugs or alcohol***						
Missing or Blank	**386 (17.9)**	118 (23.4)	268 (16.2)	6 (1.2)	3 (2.1)	3 (0.8)
*No*	**1640 (76.1)**	298 (59.1)	1342 (81.3)	454 (87.8)	102 (70.8)	352 (94.4)
*Yes*	**128 (5.9)**	88 (17.5)	40 (2.4)	57 (11.0)	39 (27.1)	18 (4.8)

The dominant group screened for risk was 10 years older in men than in women, whereas PrEP uptake was predominantly in the same age bracket (26-35 year olds). Among those at risk, 29.4% of men vs. 62.8% of women belonged to at least one target population for PrEP, yet a number of target populations were women-specific in the design of the PrEP demonstration project. In risk assessments 14% more men than women named STIs as an HIV risk factor. Sex under the influence of alcohol and drugs was mentioned by 15% more men than women, with the gender difference among PrEP initiators being even more pronounced.

Substantial risk of acquiring HIV was 12% higher (p<0.001) in men than in women in those screened for risk when only gender was taken into account, yet was found not to be statistically significant in multivariable regressions (see Table 3). For male PrEP initiators, a higher HIV risk compared to female PrEP initiators did not prove statistically significant in univariable or multivariable regressions.

**Table 3:** Gender differences in being at risk of HIV.

	Identified at risk^i^		Initiated PrEP^ii^	
	*RR (95% CI)*	*P*	*RR (95% CI)*	*P*
*Univariable*^*iii*^				
Male	1.12 (1.06 – 1.20)	<0.001	1.16 (0.95 – 1.42)	0.146
*Multivariable*^*iv*^				
Male	1.08 (0.99 – 1.19)	0.101	1.05 (0.86 – 1.28)	0.644

Abbreviations: RR=Risk Ratio; CI=confidence interval

With regard to uptake, men represented 29% of all PrEP initiators. Male participation increased only slightly from an initial 24% at the beginning of the Demonstration Project [45]. Men’s main reasons for visiting the clinics were Voluntary Counselling and Testing (VCT) (36%), Out Patient Department (OPD) (31%) and PrEP (24%). Men’s uptake among those at risk constituted 36%.

Sero-discordance was a risk factor mentioned by 21% of men screened for risk and a reason for initiating PrEP for 22% of men, yet neither univariable regression nor multivariable regression showed that men in sero-discordant relationships were more likely to initiate PrEP than men with an HIV-negative partner. Data was adjusted for the clustering of health facilities.

**Fig 1:**
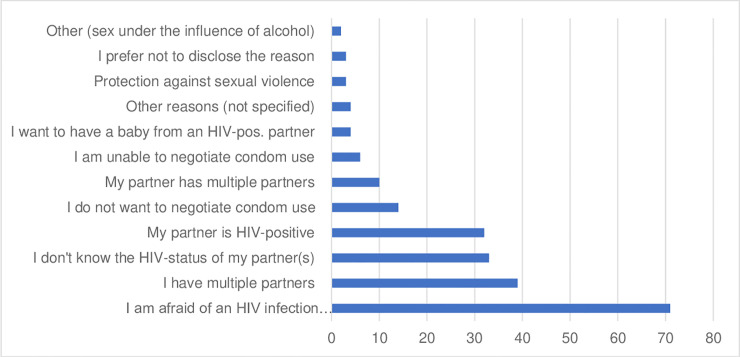
Reasons mentioned by men for taking up PrEP (Aug. 17 – Jan 19). Mutiple reasons could be given.

Risk assessment forms showed that the most important reasons for male PrEP initiation were the fear of becoming infected with HIV (32%; n=71), having multiple partners (18%; n=39), not knowing the HIV-status of a partner (15%; n=33) and living in a sero-discordant relationship (14%; n=32), see Fig 1.

These reasons were also reflected in in-depth interviews (IDIs) in terms of maintaining a negative HIV-status while having multiple partners or a sero-discordant spouse:

Respondent (R): As I have said at my age I am now sexually active and some of the girls I have sex with, I don’t know their HIV status. (…)

Interviewer (I): What motivates you to take PrEP?

R: It’s that I want to keep protected from acquiring HIV. (Male PrEP continuer, 26 yrs)

I have a wife who is taking antiretroviral pills but I am always found HIV-negative even when I am not using condoms so it dawned on me what nurses were saying: pills preventing HIV is really happening…the pills are working and I am happy with that. (Male PrEP continuer, 65 yrs)

Risk perception was, however, subjective and could be clouded by people’s own judgement. Some stakeholders and PrEP providers pointed out that many may under-, rather than overestimate the risk of getting infected with HIV:

I think individual perception of risk is generally low. So as we think on introducing PrEP, we need to be working on increasing perception of risk, because if I don’t believe that I am at risk of acquiring HIV then I won’t bother even enrolling for PrEP let alone adhering to it. (Male stakeholder, no age given)

Yet, we also found counterevidence to this statement in a man who was not sexually active at the time but felt he wanted to protect himself by taking PrEP based on past experiences.

R: I thought to myself that I have to start the pills because of my ex-wife’s HIV status. It may happen that you have a partner or wife that you may never know her HIV status and you may use a condom but it raptures so I told nurses I would like to be protected (…)

I: By the look of things, do you think you are at HIV risk now?

R: No, I am not at risk that much now. (Male PrEP continuer, 39 yrs)

Risk perception could be an important reason for initiating and declining PrEP, see Table 4.

**Table 4:** Men’s reasons for declining PrEP.

Reasons for PrEP refusal (among males identified at risk)	N	%
Need time to think about it	45	29.2
HIV is a treatable disease now	27	17.5
Don't consider myself at risk	24	15.6
Concerns about pill taking	17	11.0
Need to talk to/consult my partner first	12	7.8
I am worried about the side effects	7	4.5
Use other prevention methods	7	4.5
My family/ partner/ friends would judge me	5	3.2
Don't have time for frequent follow-up visits	4	2.6
Prefer not to disclose reason	2	1.3
I have no knowledge of PrEP	2	1.3
Don't want to get tested for HIV every 3 months	1	0.6
In a hurry, will come back some other time	1	0.6
**Total**	**154**	99,7%♦

In IDIs, PrEP decliners talked primarily about daily pill-taking as a deterrent, yet also mentioned low risk perception:

I: Why do you think you’re at low risk?

R: I’m just looking at the way my sexual activity is set-up and that most of the time I use the condom. (Male PrEP decliner, 30 yrs)

#### Testing, adherence and retention

Regular HIV testing ranked low as a reason for declining PrEP (see Table 4). IDIs shed more light on this as most decliners said they tested frequently for HIV - either alone or with their partner; some mentioned a former partner or parents living with HIV as the reason for regular HIV tests. Yet many MSW in our study saw testing as a major limitation to accessing PrEP, even for those who felt themselves at risk. They described men as mainly testing when they were sick or not testing at all either because they assumed that their HIV status was the same as their partner’s or because they were afraid of going to a clinic to test.

I: What do you think is keeping people from joining PrEP even those that are at a high risk of getting HIV?

R: Yah, it is that people do not want to get tested, yet you cannot get PrEP without testing… If you tell them, they have to get tested to get PrEP, they tell you they do not want to. That is what most people say to me. (Male PrEP continuer, 27 yrs)

R: Some don’t want to know their status. They don’t want to check. They think if they go to the clinic they will force them to test…They wish pills would just be anywhere like the condoms so they can just take and use them. (Male community leader, 35 yrs)

In addition to testing, PrEP providers saw daily pill-taking and long-term commitment as problematic:

As soon as you say it's a pill that you take every day, 50% just walk out. People just stop right there. You can watch their eyes glaze over. They start complaining – a pill every day, most people are discouraged. (Male PrEP provider, 28 yrs)

By the time we explain to them the pill, some, they start having that fear of the unknown of the pills but some they take them; some they take the first month, then after the first month we just lose them in the system. (Male PrEP provider, 41 yrs)

**Fig 2:**
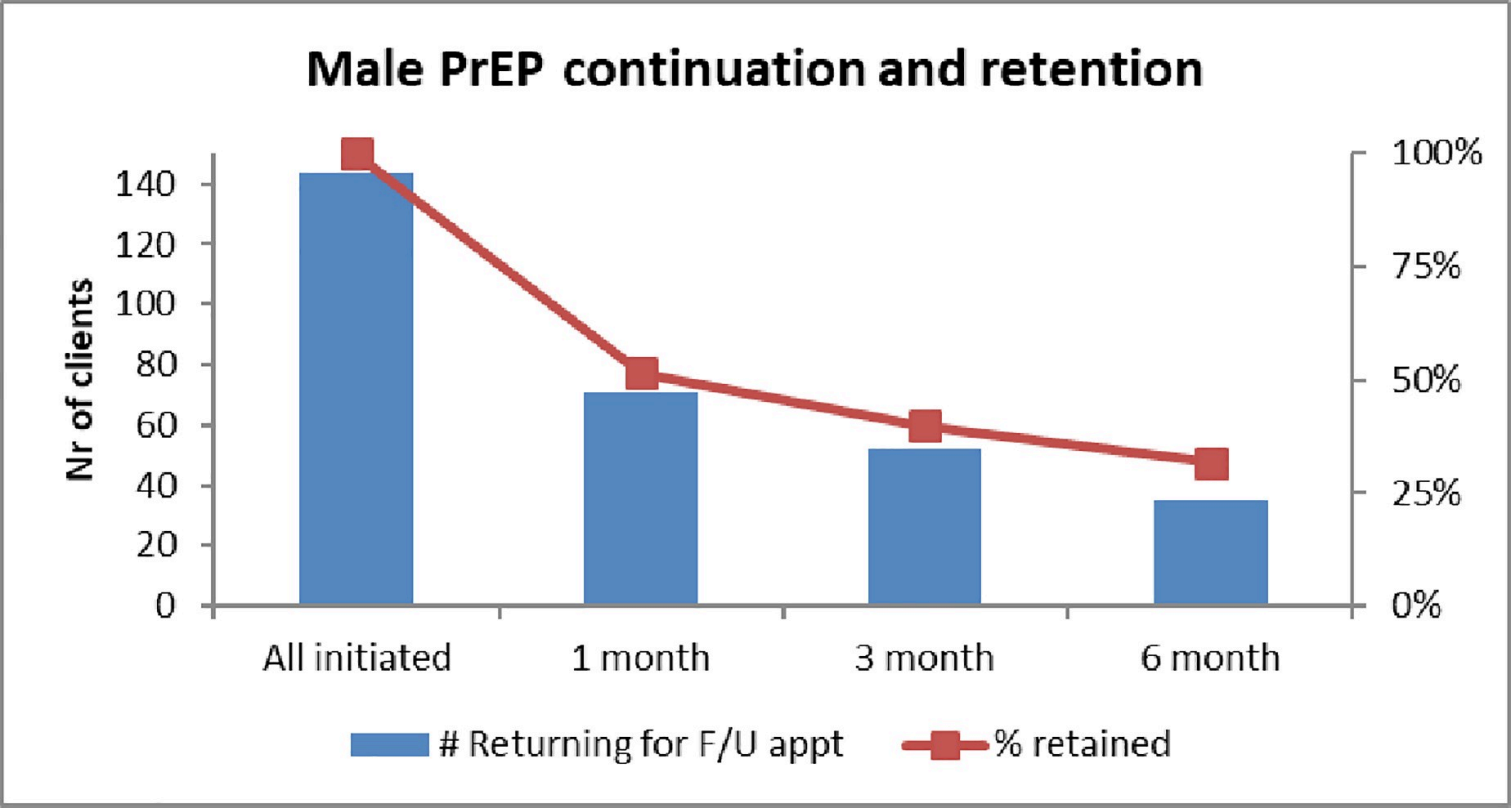
Male retention at 1,3 and 6 months. (Aug 17 – Jan 19).

Retaining male clients proved difficult: PrEP discontinuation increased from 50% after one month, to 60% after three months. After six months almost 70% of male clients had stopped taking PrEP, see Fig 2.

Some MSW across all respondent groups saw adherence and retention as a challenge in terms of busy lifestyles, working away from home or forgetting to take PrEP when socializing and drinking. When asked about men’s favored way of taking PrEP, some MSW mentioned injectables, less frequent or event-driven PrEP as a better alternative, “it must be a condom that is a pill.” (Male PrEP discontinuer, 28 yrs).

Yet those who had started or continued PrEP said that taking a pill daily had worried them *before* starting PrEP, but ultimately proved manageable. Male PrEP continuers, who had a partner on anti-retroviral treatment (ART) described coordinating the times for taking PrEP and ART pills as a useful adherence tool. Some MSW also suggested that the clinics should remind clients to stay involved in the program and to collect their PrEP pills.

The fact that PrEP and ART consist of anti-retroviral drugs (ARVs) led to some confusion about taking a prophylaxis as opposed to taking ART, which could heighten stigma for those on PrEP.

The people from rural areas do know that I take some pills but their conclusion is that I am living with HIV. (Male PrEP continuer, 30 yrs)

MSW also talked about PrEP pills rattling in the container, the same queue at health facilities for prevention and treatment, and evading close-by facilities in order to avoid meeting friends or sexual partners who may conclude that one is HIV-positive. Different messages pertaining to PrEP and ART in terms of situational versus life-long adherence confused not only PrEP-takers but “can also affect those who are HIV-positive, who are on ART. I have a cousin who started ART last month and this time has stopped” (male PrEP provider, no age given). Some MSW also worried about the effect of starting and stopping PrEP in terms of resistance and vulnerability to HIV and other infections.

While side-effects were not a major concern of male PrEP providers and stakeholders, for male PrEP discontinuers experiencing or worrying about side-effects such as having to vomit, feeling dizzy, having a temperature or feeling sleepy played a dominant role in forgoing PrEP. Men from the community taking part in FGDs as well as men engaging with PrEP also expressed concerns about serious infections (such as STIs) or a possible lowering of one’s libido “maybe the pill will kill some of my sexual feelings and even maybe give me erectile dysfunctions leading to poor sexual performance” (Male PrEP initiator, 20 yrs).

### Sexual behaviour, trust and mistrust

In almost all FGDs and IDIs, conversations about PrEP included interviewees’ experience and attitude towards condom use as another effective prevention method. A preference for PrEP played an important role for MSW in terms of better protection, compatibility with alcohol consumption, ease of usage, and additional opportunities for safe sex. A PrEP provider spoke of PrEP as the “messiah” (Male PrEP provider, 33), and some MSW viewed PrEP as a tool for more intimacy and greater pleasure:

There are some people who say that they do not feel anything when they are using condoms so when you are using this pill you can go and sleep with someone without wearing a condom and not be affected. That’s the advantage that it has, that you will feel the desired feeling during sex as you would have had your pill *(laughing);* yes that is what they say. (Male community leader, 29 yrs)

Some men also hoped that taking PrEP would increase libido, others hoped that PrEP would facilitate sexual relations and help them overcome condom-induced erection problems.

Not all of us are using condoms and especially me, when I try using it, my penis just loses erections because it doesn’t want to be covered by a condom… well I don’t know maybe it’s because I am not circumcised…I am not really sure about that, but truth is I lose erections when I use a condom (laughing) and it makes me not to go out there and have sex with other women because I have to use a condom (interviewer laughing)… maybe it’s because I am not satisfied or it could be that I wasn’t well taught about the condom because I have tried using it with my wife many times but still I lose erections. (Male PrEP initiator, 41 yrs)

The impact of alcohol intake (also in the form of the local marula beer) on forgoing condom use was mentioned by some MSW as heightening the risk of contracting HIV. MSW perceived PrEP as a more appropriate prevention tool in these situations:

This area has a lot of places that serve traditional beer (…) So they’ll be knowing that for them to be protected they must get PrEP. (Male PrEP provider, 41yrs)

While all clients were told that PrEP is an *additional* prevention method, about half of the interviewees who initiated PrEP intended to decrease or stop condom use.

It is that now that I have started taking the pills, I will go around sleeping without using even protection. I trust that I’m using PrEP” (Male PrEP initiator, 24 yrs).

Among MSW on PrEP, many interviewees stated that their condom use had remained unchanged either because they preferred comprehensive prevention or because PrEP was not effective against STIs other than HIV.

When you are wearing a bulletproof you cannot say, I won’t get hurt. What I mean is once you get the pills you still have to use condoms to get dual protection. (Male PrEP initiator, 34 yrs)

PrEP was seen by some men as creating more opportunities to engage in sexual activities without having to rely on condoms, yet no trends could be established on changes in the number of sexual partners in the qualitative data. Reasons for decreasing partners were the hope “to get settled with one partner” (Male PrEP initiator, 21 yrs) or not fully trusting PrEP’s efficacy, “fear is keeping me in check” (Male PrEP continuer, 30 yrs). Many men stated the same number of sexual partners as before.

MSW primarily saw PrEP as a tool for their own protection, yet some MSW also wanted to put the partner at ease or to protect family members, show responsibility for the children or for looking after someone sick or someone involved in an accident. A concern for others could also express itself in talking about PrEP with partner(s), family, friends and colleagues. Both partners taking PrEP led to feeling more at ease when having sexual intercourse:

You find that sometimes maybe…sometimes we have some doubts when we’re engaging in sexual intercourse. Sometimes she doesn’t trust me, saying stuff like “how can I be assured that I am your only partner who you’re sleeping with“ and stuff, so now that we are both taking PrEP, that doubt no longer appears. (Male PrEP continuer, 25 yrs)

PrEP, for some, was therefore a way of overcoming distrust in the relationship, and distrust, conversely, a reason for starting PrEP.

She (my girlfriend) is the type that is unwilling to go for HIV testing either alone or with me. Every time I bring up the idea of testing, she postpones and so this has gotten me dead-worried and concerned on why she is doing this. So this weird act of hers has got me not trusting her that much. So upon hearing about this pill, I was somehow relieved that at least even if my girlfriend is up to some kind of promiscuous practices, I will still be safe from contracting HIV. (…) I don’t trust myself, I don’t trust my girlfriend, and I just don’t trust anyone. We’re humans and make mistakes, so I think once I am initiated on PrEP, I’ll take it every day so I can be protected for the rest of my life. (Male PrEP initiator, 20 yrs)

In addition to the partner not wanting to test for HIV, men mentioned distance through work or living arrangements as reasons for mistrust. At the same time, men saw the need to consult the partner *prior* to starting PrEP (the fifth most important reason for men to decline PrEP when it was offered to them on the same day, see Table 4). Conversely, some men viewed conversations around PrEP as creating problems in the relationship

I live with my partner and we never use any kind of protection in the hope that we are both faithful to each other. And if I were to take the pill then how would I explain that to her? (Male PrEP decliner, 34 yrs)

Trust and mis- or distrust as well as being able or unable to talk about PrEP with the partner played an important role for starting or declining PrEP.

### PrEP education and service delivery

In IDIs and FGDs MSW expressed concern about PrEP education and service delivery in health clinics, a location largely avoided by men.

A problem is that I don’t know what we are doing as men because if you can look at it carefully no matter where you go, the men do not want to go to the hospital. They want to go when they are being pushed in a wheelbarrow. (Male community leader, no age given)

It’s hard for us men to go to the clinic. We naturally have a fear of hospitals. We are even afraid of doing HIV testing. (Male FGD participant, 31 yrs)

Men commented that offering PrEP and PrEP information in health facilities would restrict its benefit to those who customarily frequented clinics.

If you are rolling out PrEP in facilities – you are doing the same old mistakes you were doing in the past. So how could you do it? I don't have a proper answer. Can you take treatment to people? I don't know. But you definitely have to do different things. (Male stakeholder, 32 yrs)

In terms of PrEP education, the majority of men taking part in IDIs or FGDs favored a decentralized model.

People shouldn’t wait to get to the clinic to then learn about the pill, they should know about it at community level cos it’s not like everyone comes to the clinic. (Male PrEP decliner, 30 yrs)

In order to make PrEP known, men suggested the following approaches: PrEP education in the community, at the chief’s place, at schools, at cattle plunge dips, at taxi ranks and bus stations, in drinking places, in churches, through local organizations, groups, peer educators or rural health motivators, at soccer matches or by going door-to-door. They suggested to place flyers or other information material in shops and offices and publicize PrEP through printed media, radio and television.

While some MSW experienced no problems with PrEP service delivery, others mentioned clinic-based barriers such as the attitude of staff, time-consuming hospital procedures, transport issues, and the necessity for regular clinic visits that could not be easily combined with work schedules.

I cannot queue in a clinic for the whole day and then not get my PrEP. Who has time for that? I cannot spend all my money coming to Mbabane to get my PrEP. I cannot do these things. (Male PrEP continuer, no age given)

I: The whole process takes about an hour and a half.

R: It takes?

I: An hour and a half.

R: (Laughs) Ok. For men you are not likely to see higher uptake based on the process… Men want a quick thing, I don’t know but I don’t see men rushing to it based on the long process to be there. (Male stakeholder, no age given)

A male stakeholder also raised the issue of the health set-up: “there’s not a toilet for men in these public health facilities. It should be for everyone” (32 yrs).

In order to attract more men, MSW across all categories of interviewees and participants in FGDs recommended changes for PrEP service delivery such as expedited services, a separate queue and separate clinic rooms for PrEP, more confidentiality, more male health personnel, a section for men at the clinic, separate male clinics or mobile clinics, integrated health services, being treated with respect, and incentives for men in terms of food, T-shirts or refreshments.

They [women] have the time and the patience. Men won’t. They don’t want this. They want you to tell them and give it to them, with food and gifts. (Male PrEP continuer, no age given)

Despite few men utilizing health facilities, most community leaders favored to maintain a decentralized hospital and clinic-based delivery approach for PrEP, as this would ensure PrEP delivery by qualified health personnel. Yet a clinic approach was not favored by all MSW. Some suggested alternative PrEP delivery models through mobile services, schools, shops, workplaces, door-to-door or even through the chief or local councilors:

…the problem is that men do not like to go to hospital so it will be difficult for them to get PrEP easily…Maybe the rural health motivators, because they find the men at home, so if we can give those that go to the homes to go with PrEP. It could also be *Bucopho* [local councilor], if maybe they have been trained on PrEP. (Male community leader, 35 yrs)

The technicalities of how this may happen was not further elaborated, yet MSW recommended to decentralize information and service delivery of PrEP, to use local clinics and to test new community approaches in an attempt to make PrEP more accessible for men.

## Discussion

Our study, nested within a general population PrEP demonstration project in Eswatini, shows that MSW at risk of an HIV infection and male PrEP initiators mainly belonged to the age group 26-35 years, had multiple partners and/or did not cohabit with their partner. PrEP initiation by MSW was mainly facilitated by their own risk perception, by a desire for better HIV protection, practicing unprotected sex or having a sero-discordant partner or a partner of unknown HIV-status. Mistrust in the partner(s), preference for PrEP over condom use and the hope of facilitated sexual relations and/or performance, consuming alcohol or having STIs and a concern for the family were further reasons to initiate PrEP. MSW’s barriers to PrEP uptake and continuation included needing time to think about PrEP, a low risk perception, daily pill-taking, anticipated or experienced side-effects and facility-based PrEP education and service delivery.

Analogous to MSM studies in the United States [46–48] male PrEP uptake was low in Eswatini (29% of the men identified as being at substantial risk of an HIV infection). Clinic-based HIV testing, information and PrEP service delivery appeared to be restricting factors; this resonates with other PrEP, HIV and health studies that show a general reluctance by men to use health facilities [49–52]. Suggestions made by MSW in our study can be seen as an adaptation of ART delivery models to PrEP [53] in terms of male-friendly, integrated and community services such as mobile and door-to-door PrEP. MSW’s recommendations for PrEP education in places where men congregate and PrEP delivery through key people (chiefs, councilors, local health workers) or key institutions (schools, workplaces) would have to be trialed to determine appropriateness regarding regular HIV-testing and confidentiality. MSW’s preference for less frequent PrEP such as injectables has also been depicted in women-focused studies as aiding adherence [54,55], while on-demand oral PrEP has so far been trialed and proved effective for MSM [56], yet could also widen MSW’s PrEP choices. MSW further suggested to include more counselling and client follow-up to motivate men to stay engaged with PrEP.

Our study showed that similarities between daily PrEP and ART had a positive effect on adherence in joint pill-taking of sero-discordant couples, described by Ware et al as a strategy to overcoming potential relationship conflicts [57]. Similarities between PrEP and ART could, however, also lead to confusion: PrEP use hinges on perceived risk situations and preferences for a particular prevention tool. Stopping and starting pill-taking is to be expected with PrEP, and some scholars speak about PrEP as a “prevention-effective” strategy [53,58], i.e. being used as long as the risk exists; ART, by contrast, is a life-long treatment strategy [3]. High rates of men dropping out of PrEP programs, such as in the Eswatini demonstration project, are therefore not a problematic phenomenon as such, yet the spill-over of the stop and start behavior of PrEP clients to ART clients, as reported by one of the PrEP providers in Eswatini, is worrying and will have to be addressed.

We also noted MSW’s concerns of being mistaken for a person living with HIV in queueing together at clinics or being seen to take pills, which are also used for ART. Studies on MSM, by contrast, refer to stigma associated with more “promiscuous” sexual behavior [59,60]. This type of stigma was hardly mentioned by MSW in our study, yet MSW talked about the expectation or concern that PrEP may lead to greater risk-taking behavior. Risk compensation has been a major concern for all HIV prevention methods from condoms, male circumcision, vaccines and microbicides to PrEP [61–63]. While a recent PrEP review of population groups [64] and PrEP clinical trials and demonstration projects [65–69] showed no marked difference in sexual behavior as a result of PrEP, a systematic MSM PrEP review demonstrated an increase in STIs and less frequent condom use [63]. In addition, studies on men often emphasize multiple partners and sexual prowess [70–72] implying that PrEP may lead to ‘sexual disinhibition’ [73]. Our qualitative data, by contrast, shows that most MSW practiced the same sexual behavior as before in terms of the number of sexual partners. The forgoing of condoms by about half of the male PrEP initiators interviewed needs to be seen in relation to condom use prior to PrEP use, which may not have been consistent, as the British PROUD study documented for MSM [74]. A recent MSM study among IPERGAY participants described MSM’s experience of more pleasurable and serene sexual intercourse as they did not have to deal with the material barrier of a condom or the anxiety of putting it on correctly while interrupting intercourse [75]. Some MSW in Eswatini shared these sentiments and even hoped that PrEP might increase libido, yet their response also included concerns that PrEP may lead to erectile or relationship problems.

Our study revealed that MSW are a risk group in their own right who demonstrated interest in PrEP as a novel HIV prevention method. In order to strengthen MSW’s uptake and use of PrEP, risk perception would, however, have to increase and PrEP information and service delivery would have to be provided in a male-friendly manner. While other qualitative PrEP studies have focused on PrEP acceptability [23–27,31], views of MSW as part of ethnic or migrant communities [29,30], or sero-discordant couples’ experience with adherence and side-effects [32, 33], our study has depicted MSW’s attitudes and experiences of PrEP in terms of individual, social and sexual behavior and men’s use of clinics in a wider population study.

## Conclusion

Despite MSW’s general vulnerability towards HIV in generalized epidemics, PrEP studies on men have mainly focused on MSM or men in sero-discordant relationships. Our study has contributed to the scarce PrEP literature on a general MSW population from the perspective of male clients as well as male stakeholders, PrEP providers, community leaders and members. MSW’s risk of contracting HIV, low PrEP uptake and experiences of PrEP in Eswatini require PrEP programs to be tailored to men’s needs in terms of accessibility and delivery processes. Further and larger PrEP intervention studies are needed to test MSW-favored PrEP education and service delivery in communities and at places where men congregate. More MSW studies would also deepen our understanding of MSW’s experiences of PrEP in terms of enabling and impeding factors in order to advance a population roll-out of PrEP that benefits MSW.

## Supporting information

S1 File (PDF)

S2 File (DOCX)

S3 File (DOCX)

S4 File (DOCX)

S5 File (DOCX)

S6 File (DOCX)

S7 File (DOCX)

S8 File (DOCX)

S9 File (DOCX)

S10 File (DOCX)

S11 File (DOCX)

S12 File (DOCX)

S13 File (DOCX)
