# Supplementary material for: “It’s hard for us men to go to the clinic. We naturally have a fear of hospitals.” Men’s risk perceptions, experiences and program preferences for PrEP: A mixed methods study in Eswatini
Source: PLoS One. 2020 Sep 23;15(9):e0237427. doi: 10.1371/journal.pone.0237427 (PMC7510987; doi:10.1371/journal.pone.0237427)
Supplement: S1 File — (PDF) [file pone.0237427.s001.pdf]

PrEP for HIV Prevention: Part A Risk assessment

Facility name: _________________

HISCC PILOT END DATE: 31/03/2019 Ministry of Health

[insert serial number]

Date: DD MM YYYY

Consent for screening: ☐ Yes → ☐ First time PrEP Screening ☐ Repeat screening
☐ No

Sex: ☐ Male ☐ Female ☐ Transgender

DOB: DD MM YYYY

Reason for visit: ☐ PrEP ☐ VCT ☐ OPD ☐ STI treatment ☐ ANC ☐ PNC
☐ FP ☐ Other, _________________ ☐ Referral # _________________

Relationship status: ☐ Single, no relationship ☐ One partner, living together
☐ Multiple partners ☐ One partner, not living together

Partner HIV status: ☐ Negative ☐ Positive ☐ Unknown ☐ No answer

If partner HIV-positive: ☐ Partner on ART ☐ Partner **NOT** on ART ☐ Unknown partner ART status

Education: ☐ None ☐ Secondary
☐ Primary ☐ Tertiary

HIV test date: DD MM YYYY ☐ Non-reactive ☐ Indeterminate ☐ Reactive → ☐ Linked to ART

HTS register #: _____________

Perceived risk: On a scale of 1-5, how high does the client perceive his/ her risk to get HIV. Circle the correct number.
1: No risk 2: Low risk 3: Some risk 4: High risk 5: Very high risk

In the past SIX months:		
1. Have you had unprotected (condom-less) sex?	☐ Yes	☐ No
2. Have you had sex with partners who are HIV positive or whose HIV status you did not know?	☐ Yes	☐ No
3. Have you had a sexually transmitted infection?	☐ Yes	☐ No
4. Have you been using post-exposure prophylaxis (PEP)?	☐ Yes	☐ No
5. Have you had sex under the influence of alcohol and/or drugs?	☐ Yes	☐ No
6. Have you experienced or do you expect any situations which you consider to be risky for acquiring HIV? If yes, specify: ____	☐ Yes	☐ No

If known, indicate if the client belongs to any of the following target populations (tick any that apply):

Young woman 16 – 25 years	☐ Yes	☐ No	
In sero-discordant relationship:	☐ Yes	☐ No	☐ Unknown
Sex worker:	☐ Yes	☐ No	☐ Unknown
MSM:	☐ Yes	☐ No	☐ Unknown
Client with current STI:	☐ Yes	☐ No	☐ Unknown
Pregnant:	☐ Yes	**EDD:** DD MM YYYY	
	☐ No	**LMP:** DD MM YYYY	
Lactating:	☐ Yes	☐ No	

Other at risk as per risk assessment:
☐ Yes ☐ No

If yes, specify:

Comments:____
____

Conclusion:

☐ Client at substantial risk for HIV infection and interested in PrEP
→ Continue with eligibility assessment on next page

☐ Client at substantial risk for HIV infection and **NO** interest in PrEP
→ Discuss, offer and/ or refer for other HIV prevention services

☐ Client not at substantial risk for HIV infection
→ Discuss, offer and/ or refer for other HIV prevention services

Provided counselling on:
- ☐ Condoms
- ☐ VMMC → ☐ Referred for VMMC
- ☐ Delayed sexual debut
- ☐ Reducing # of sexual partners
- ☐ STIs
- ☐ Partner testing
- ☐ Test and Start
- ☐ Other, specify: _______________

Initial & Date (Clinic Staff): ___________________________ Initial & Date (Data Staff): _____________________________

PrEP for HIV Prevention: Part B Eligibility assessment

Acute HIV Infection (AHI)

In the past 3 days have you had any of the following symptoms?

Sore throat	☐ Yes	☐ No
Fever	☐ Yes	☐ No
Night sweats	☐ Yes	☐ No
Swollen glands	☐ Yes	☐ No
Mouth ulcers	☐ Yes	☐ No
Headache	☐ Yes	☐ No
Rash	☐ Yes	☐ No
Generalized body pain	☐ Yes	☐ No
Intense fatigue	☐ Yes	☐ No

Possible exposure to HIV in the last **14** days: ☐ Yes ☐ No

☐ ≥1 symptom + possible exposure
→ Suspect AHI, defer PrEP initiation

☐ ≥1 symptom + no exposure
→ Differential diagnosis: ________________________

TB screening: ☐ Neg. ☐ Pos. ☐ Active TB

STI symptom screening

In the past 3 days have you had any symptoms of an STI?

Genital sore or ulcer	☐ Yes	☐ No
Vaginal/penile/anal discharge	☐ Yes	☐ No
Vulval/penile itching /burning	☐ Yes	☐ No
Lower abdominal pain	☐ Yes	☐ No
Scrotal swelling	☐ Yes	☐ No
Inguinal bubo	☐ Yes	☐ No

Differential diagnosis: _________________________

RPR / Syphilis (if symptomatic) ☐ Yes ☐ No

Date tested: DD MM YYYY

Result: ☐ Non-reactive ☐ Reactive → ☐ Rx given

Hepatitis B test and vaccination

HBsAg test date: DD MM YYYY Result: ☐ Neg. ☐ Pos.

If HBsAg positive: AST result_____ ALT result_____

Management______________________________________

Vaccination: ☐ Not done ☐ Reported ☐ Documented

Known NCDs:

HPT: ☐ Yes ☐ No

BP: ____________

DM: ☐ Yes ☐ No

Serum creatinine

Date sample drawn: DD MM YYYY Result: _____ µmol/ L

Age : _________ years CrCl: _______mL/min

Weight: _________ kg

Male: $\frac{(140 - \text{Age}) \times \text{weight in kg} \times 1.23}{\text{Serum creatinine (in µmol/L)}}$

Female: $\frac{(140 - \text{Age}) \times \text{weight in kg} \times 1.04}{\text{Serum creatinine (in µmol/L)}}$

Eligibility checklist (tick all that apply)		
1. Participant is ≥16 years	☐ Yes	☐ No
2. HIV test is non-reactive	☐ Yes	☐ No
3. At substantial risk for HIV infection	☐ Yes	☐ No
4. Do not suspect acute HIV infection	☐ Yes	☐ No
5. Baseline creatinine taken	☐ Yes	☐ No
6. Baseline HBsAg taken	☐ Yes	☐ No
7. Participant is > 40 kg	☐ Yes	☐ No
8. Participant is willing/ able to come of follow up appointments	☐ Yes	☐ No
9. No contraindications to TDF (see guidelines)	☐ Yes	☐ No

Eligible for PrEP: ☐ Yes ☐ No ☐ Pending

Informed consent signed: ☐ Yes ☐ No

PrEP initiation: ☐ Yes ☐ No ☐ Deferred

If PrEP deferred next review date: DD MM YYYY

Other services offered/ provided:

Comments:____

____

Initial & Date (Clinic Staff): ____________________________ Initial & Date (Data Staff): ____________________________
