# Supplementary material for: “It’s hard for us men to go to the clinic. We naturally have a fear of hospitals.” Men’s risk perceptions, experiences and program preferences for PrEP: A mixed methods study in Eswatini
Source: PLoS One. 2020 Sep 23;15(9):e0237427. doi: 10.1371/journal.pone.0237427 (PMC7510987; doi:10.1371/journal.pone.0237427)
Supplement: S2 File — (DOCX) [file pone.0237427.s002.docx]

FOCUS GROUP DISCUSSION GUIDE

As we went over in the consent, all of the information you provide will be kept confidential and your names will not be recorded. Just as a reminder our discussion will probably last around 60 minutes. Some of the questions I will ask you may not want to answer and that is fine. Remember that your participation is completely voluntary. Also please keep in mind that there are no right or wrong answers, I am interested in anything you can share with me. Does anyone have any questions before we begin? May I start the recording? *[Start recording]*

**Good [afternoon/morning] thank you for participating today**! The Ministry of Health has recently started to pilot a new HIV prevention method called PrEP in some selected facilities in the country. PrEP stands for Pre-exposure prophylaxis and is a HIV prevention method. It is recommended for people who are at very high risk of contracting HIV. PrEP is a pill that needs to be taken everyday in order for it to work properly. When taken daily, PrEP has been shown to reduce the risk of HIV infection in people who are at high risk by up to 92%. There are, however, side effects that may be associated with taking PrEP. PrEP is much less effective if it is not taken consistently. People who use PrEP must commit to taking the drug every day and seeing their health care provider for follow-up every 3 months. PREP does not prevent pregnancy or other sexually transmitted infections.

I have asked you to meet with me in the hopes of learning more about the best way to promote this new HIV prevention method and educate people who could potentially benefit from PrEP. We will use the information learned from this group discussion to design a feasible, understandable, effective and culturally sensitive PrEP promotion package (PPP) that will be tested in the health facilities participating in the study.

Each of you was invited to participate in this discussion as you are all representing different groups of the community and your opinions can inform us to develop the best PPP. Your comments and opinions will be strictly confidential. We will be taking notes and also recording our conversations so that we can accurately capture and report your views. Your comments will be combined with those from other meetings as well as information gathered from interviews with clients at facilities, health care workers and policy makers and implementers.

| 1. We're going to be talking today a lot about HIV and HIV prevention campaigns. To begin, how do you think HIV is viewed in Swaziland today? How does this compare with the past? |
| --- |
| 1. Think of a health campaign around HIV prevention that, in your opinion or in the opinion of others in the community, was done well. It could be something that you have heard on the radio, read on a poster, seen in a dramatized play, learned about from a friend or during a community event. Think broadly. Do you have one in mind (interviewer pauses for confirmation) Good. Now please tell me about that. What makes you feel that this communication message was well done? What did people in the community like about the message? What made it effective? |
| 1. Now, think of a communication initiative around HIV prevention that, in your opinion or in that of others, was not done well. What makes you feel that this communication message was not done well? What did people in the community not like about the message or the method? |
| 1. How and where do you and others in the community normally receive information around HIV prevention services? |
| 1. Where would you and others in this community like to receive information about HIV prevention services? Why? |
| 1. What can be done to promote access and utilization of HIV prevention services? *(Probe for men/ women/young people/ couples/ key populations)* |
| 1. “Now I would like to show you some educational materials about PrEP that you might or might not have seen before. (Interviewer shows the current MoH PrEP material to the respondent). Please tell me some words that come to your mind when you see this (INSERT WORD e.g. poster, flyer, palm card). There are no right or wrong words; I am looking to learn from your first impressions and thoughts. All thoughts are welcome 2. PROBES- Is there anything you like about this? Please tell me more about that. 3. PROBES- Is there anything you don’t like about this. Please tell me more about that. 4. PROBES- What do you read as the main message from this? Please tell me more about that. PROBES- If you would be able to change this material, is there anything you would change/ add. Please explain? |
| 1. Thank you for your thoughts about this flyer/ poster/palm card. Thinking back to the communication message you mentioned you liked in the beginning, do you think similar material/ methods can be adopted for PrEP? Why or why not? |
| 1. Do you talk about health messages with your friends and family or others you know? How does the conversation typically go? |
| 1. Do you think you would be comfortable to talk about PrEP with your friends, family or other people you know? Please explain? |
| 1. We are trying to make the most effective, informative PrEP messaging possible and to make it easier for people to access PrEP. Can you think of anything else that we should consider in order to inform and educate more people about PrEP? |
| 1. Are there other things you would like to say before we wind up? |
