# Supplementary material for: “It’s hard for us men to go to the clinic. We naturally have a fear of hospitals.” Men’s risk perceptions, experiences and program preferences for PrEP: A mixed methods study in Eswatini
Source: PLoS One. 2020 Sep 23;15(9):e0237427. doi: 10.1371/journal.pone.0237427 (PMC7510987; doi:10.1371/journal.pone.0237427)
Supplement: S3 File — (DOCX) [file pone.0237427.s003.docx]

**TOOL – IN DEPTH CLIENT INTERVIEWS – PREP UPTAKE (NEW CLIENT)**

**Client experiences with uptake and use of HIV pre-exposure prophylaxis in Swaziland**

As we went over in the consent, all of the information you provide will be kept confidential. Just as a reminder our interview will probably last around 45-60 minutes. Do you have any questions before we begin? May I start the recording? *[Start recording]*

**Good [afternoon/morning] thank you for participating today**! I have asked you to meet with me in the hopes of learning more about your experience with pre-exposure prophylaxis (PrEP) and themes related to how to improve your experience learning about, accessing and taking PrEP. Some of the questions I will ask you may not want to answer and that is fine. Remember that your answers are confidential and participation is completely voluntary. Also please keep in mind that there are no right or wrong answers, I am interested in anything you can share with me.

Questions for participants who initiated PrEP:

| **Question** |
| --- |
| 1. As I mentioned earlier the main focus of our discussion is PrEP. Please think of the first time you heard about PrEP. Do you remember the first time you heard about it? (Pause, await respondent) Okay, good.    1. Keeping in mind that there are no right or wrong answers, in your understanding, what is PrEP?       1. What would be a name that you would give PrEP?    2. Why would a person take PrEP?    3. Why would a person not take PrEP?    4. Who is PrEP for?    5. What else do you know about PrEP? |
| Now please walk me through your story from when you heard about PrEP until now. If you don’t mind, I’ll interrupt sometimes to get more details. When and where did you first hear about PrEP?   - - 1. What did you think when you heard about this pill?     2. Were there any things that you wondered about when you first heard about this pill? If you could have had more information, what would you have liked to know?   What made you think that PrEP made sense for you?  What made you think that PrEP would be effective?  What makes you feel good about starting on PrEP?  What makes you feel concerned or worried about starting PrEP? |
| 1. Did you have any hesitations about starting PrEP? How did you consider hesitations versus reasons to start taking PrEP? |
| 1. When you need to collect your pills for PrEP, what will be the steps you need to take? 2. Are there some things that you can imagine will make it hard to collect the pills? 3. Are there some things that will make it easy to collect the pills? 4. What do you think the health facility or others who design health promotion programs could do to make it easier for you to collect your PrEP pills? |
| 1. There are a lot of reasons that people take or don’t take pills even if it is recommended. Sometimes, even if people are at high risk for HIV they do not want to take PrEP. What do you think would be useful to improve getting people who are at risk for HIV to take PrEP? |
| There are also a lot of reasons that people take or don’t take medicine as often as the health provider recommends. When it comes to PrEP, what do you think will make it hard or easy for you (and/or others you know) to take the pill as often as the provider recommends? What do you think would be useful to improve this for you or others? |
| 1. Now, I would like to discuss something a bit more intimate. Please remember that all information you share is confidential and will only be shared with the research team for purposes of improving access to PrEP. Ok? (pause). I would like to talk about your intimate life in relation to PrEP (pause). How do you think PrEP may affect your sexual life? 2. Has it affected or might it affect, whether or how you discuss HIV status with partners? 3. How might it effect whether you use condoms? 4. How might it effect on the number of sexual partners you may have? |
| 1. Do you think PrEP is a topic that men and women in Swaziland feel comfortable discussing with friends and/or partners? Why or why not? |
| 1. Do you know anyone else who is currently taking PrEP? How was their experience starting on PrEP different or similar to yours? How did you and this person come to start talking about PrEP? |
| 1. Thinking of a friend you know who is on PrEP, what do you think may be some of the challenges they face when |
| 1. Thinking about Swaziland and your community, what are some things that would make it hard for people like you and people you know to get PrEP? How could things be changed to make it easier for you and others in Swaziland to get and routinely take PrEP? |
| 1. Now I would like to show you some materials about PrEP that you might or might not have seen before. (Interviewer shows the standard MoH PrEP material to the respondent) 2. Please tell me some words that come to your mind when you see this flyer/poster. There are no right or wrong words; I am looking to learn from your first impressions and thoughts. All thoughts are welcome.    - 1. PROBES- Is there anything you like about this? Please tell me more about that.      2. PROBES- Is there anything you don’t like about this. Please tell me more about that.      3. PROBES- What do you read as the main message from this? Please tell me more about that.      4. PROBES- What message is missing from this flyer? What more would you like to know?      5. PROBES- If you would be able to change this flyer/ poster/palm card, what would you change (if at all)? |
| 1. Thank you for your thoughts about this. Now I would like you to think about other ways that you have learned about other health matters. Do you have a health campaign like for malaria or HIV or TB in mind where you felt like you learned something (interviewer pauses for confirmation)? Good. Now please tell me about that.    1. PROBES- What did you like about (INSERT RESPONDENTS FAVORITE MESSAGING MECHANISM)    2. PROBES- Do you think we could do something similar for PrEP messaging. If yes, how would/ should it be tailored to PrEP. If not, why do you think PrEP doesn’t fit with (it)?    3. PROBES- Do you talk about health messages with your friends, family or others you know? Please explain with whom?    4. PROBES- We are trying to make the most effective, informative PrEP messaging possible and to make it easier for people to access PrEP. Can you think of anything else that we should consider in order to make it possible? |
| 1. Now let’s think about the future. How do you envision plans in the coming months in relation to PrEP, if at all? Some people who start PrEP view it as a drug that they will take forever, others view it differently. What is your view of how you will take PrEP in the coming months?   What are some life events or other factors that would influence whether you would continue to take PrEP in the future?   - - 1. What are some circumstances that you could imagine would make it very hard for you to stay on PrEP?     2. What could people who oversee health interventions do to help you over come this challenge? |
| 1. Is there a time earlier in life when, looking back now, you wish you would have had access to PrEP? Please tell me about it. |
| 1. To conclude, what would be your recommendation to improve your experience with PrEP? |
| 1. What would you recommend to improve others’ experience and access to PrEP? |
| 1. And finally, is there anything that I didn’t ask you that I should have asked you? |
| 1. Is there anything else that you would like to add? |
