# Supplementary material for: “It’s hard for us men to go to the clinic. We naturally have a fear of hospitals.” Men’s risk perceptions, experiences and program preferences for PrEP: A mixed methods study in Eswatini
Source: PLoS One. 2020 Sep 23;15(9):e0237427. doi: 10.1371/journal.pone.0237427 (PMC7510987; doi:10.1371/journal.pone.0237427)
Supplement: S4 File — (DOCX) [file pone.0237427.s004.docx]

**TOOL – IN DEPTH CLIENT INTERVIEWS – PREP UPTAKE (NEW CLIENT)**

Client experiences with uptake and use of HIV pre-exposure prophylaxis in Swaziland

 Njengoba sikhulumisene masicela imvumo yakho, konkhe lotasitjela kona kutawugcinwa kuyimfihlo. Kukukhumbuta, lokucocisana kwetfu kutawutsatsa sikhatsi lesibekiselwa kumizuzu lengu 45 kuya ku 60. Ngabe kukhona yini imibuto lonayo singakacali? Ngicela kucala kurekhoda ngemvumo yakho?

**__________________________________________________________________________________**

 Sawubona... Siyabonga kutsi uvume kuba yincenye yalokucocisana kwetfu namuhla. Ngicele kutsi sihlangane namuhla ngenhloso yekutfola lwati lolubanti ngaloke wahlangabetana nako ngekunatsa emaphilisi ngenhloso yekuvikela kutseleleka ligciwane leHIV, lokubitwa nge pre-exposure prophylaxis (PrEP) nako konkhe lokuhambisana nelwati lwakho ngekufundza, kutfola, nekutsatsa lamaphilisi e-PrEP. Kungenteka kube nemibuto longafisi kuyiphendvula, naloko akunankinga. Khumbula kutsi awukaphoceleleki kungenela lokucocisana kwemibuto. Ngicela ukhumbule futsi kutsi kute timphendvulo letikahle noma letingasiko kahle. Ngifise kuva nje konkhe longakuveta noma lokucabangako.

Questions for participants who initiated PrEP:

| Question |
| --- |
| 1. Njengoba sike sakhuluma phambilini, kucocisana kwetfu kutawuba nge PrEP. Ngicela ucabange ngalesikhatsi uva nge PrEP kwekucala. 2. Usakhumbula yini ngesikhatsi uva nga PrEP kwekucala ngca? 3. Kube semcondvweni wakho kutsi kute imphendvulo lekahle noma lekabi. Ngekucondza kwakho ngabe yini PrEP? 4. Yini ligama leSiswati longalinika  PrEP 5. Ngabe umuntfu angayitsatselani nje I PrEP? 6. Ngabe umuntfu angete ayitsatselani I PrEP? 7. I PrEP ilungela bani? 8. Yini lokunye lokwatiko nge PrEP? |
| 1. Nyalo ngicela ungicocele ngendzaba yakho kusukela weva nge PrEP kuze kube ngunyalo. Nangabe ute inkinga ngitocela kuhle ngikumisa kutovisisa kabanti 2. Kunini, phindze kukuphi lapho weva khona nga PrEP kwekucala? 3. Wacabangani nawuva ngePrEP? 4. Tikhona yini letinye tintfo lowacabanga ngato nawucala kuva ngaleliphilisi? 5. Yini lokwakwenta ucabange kutsi PrEP uyakulungela? 6. Yini lokwakwenta ucabange kutsi I PrEP itakusebentela? 7. Yini lokukwenta utive ukhululekile/ujabula ngekucala iPrEP? 8. Yini lekwenta ube nekukhatsateka ngekucala iPrEP? |
| 1. Bekukhona yini kungabata tsite ngekucala iPrEP? 2. Wabese ukhetsa njani kulokungabata kanye naletizatfu betikwenta ufune kucala iPrEP? |
| 1. Tinyatselo tini lotilandzelako nawuyolandza emaphilisi akho ePrEP? 2. Kukhona yini tintfo locabanga kutsi titawenta kube lukhuni kuyolandza emaphilisi? 3. ArKukhona yini tintfo locabanga kutsi kutawenta kube lula kuyolandza emaphilisi? 4. Tintfo tini letingentiwa litiko letemphilo noma tinsita letakha tinhlelo tetemphilo kutsi kube melula kubantfu labafana nawe kungenela baphindze bahlale kuleluhlelo lwe PrEP? |
| 1. Tinengi tizatfu letenta bantfu batsatse noma bangatsatsi emaphilisi noma ngabe bakhutsatwe kutsi bawatsatse. Ngisho noma ngabe basengotini lenkhulu yekutfola ligciwane le HIV labanye abafuni kucala iPrEP. 2. Ucabanga kutsi yini lokungasita kukhanga bantfu labasengotini ye HIV bacale iPrEP? |
| 1. Tinengi tizatfu letenta bantfu batsatse noma bangatsatsi emaphilisi abo njengoba betemphilo bakhutsata. Nasiku PrEP yini locabanga kutsi kutawenta kube matima noma kube lula kutsi wena noma labanye lobatiko batsatse I PrEP ngendlela betemphilo labakhutsata ngayo? 2. Ucabanga kutsi yini lokungasita kukhutsata loku kuwe nakulabanye? |
| 1. Nyalo ngitocela sikhulume ngetelicansi. Khumbula kutsi timphendvulo takho tiyimfihlo titawubonwa ngulabenta lucwaningo kuze kutsi kutfolakala kwe PrEP kutfutfuke. Uyeva mosi? (Pause). 2. Ngicela sikhulume ngetelicansi kanye ne PrEP (pause). 3. Ucabanga kutsi I PrEP ingayitsikabeta kanjani imphilo yakho yetelicansi? 4. Kukutsikabetile yini noma kungakutsikabeta kucocisana nalotsandzana naye/nabo ngesimo sakho sengati? 5. Ucabanga kutsi kungakutsikabeta yini kusebentisa kwakho icondom? 6. Ucabanga kutsi kungayishintja kanjani inombolo yebantfu loya nabo ecansini? |
| 1. Kuyintfo leyindzabamlonyeni yini le PrEP eveni laKaNgwane kutsi bangani netingani tingakhona kucoca ngayo ngalokusebaleni? 2. Uma kwenteka, kwentiwa yini futsi uma kungenteki, kubangelwa yini? |
| 1. Ukhona yini lomatiko lanatsa I PrEP? 2. Imivo yabo yekucala PrEP iyafana yini noma ihlukile kunaleyakho? 3. Nacala njani kucoca nge PrEP nalona muntfu? |
| 1. Cabanga umngani lomatiko loku PrEP, ucabanga kutsi ngabe yini tinkinga lahlangabetana nato nabazama kunatsa I PrEP? 2. Ngicela usho kutsi lenkinga yakhe ihlupha bonkhe bantfu noma umngani wakho kuphela? |
| 1. Nawucabanga ngelive laKaNgwane noma ngemmango wangakini, tintfo tini letingenta kube lukhuni kutsi bantfu labafana nawe bakhone kungenela loluhlelo lwe PrEP. Tingashintjwa njani leto tintfo kuze kutsi kube melula kutsi bantfu KaNgwane labafana nawe bakhone kungenela loluhlelo lwe PrEP? |
| 1. Nyalo ngitawutsandza kukukhombisa natintfo letiphatselene naPrEP lokungenteka kutsi ukewakubona phambilini noma awukake ukubone. (lobutako atjengise lophendvulako letintfo takaPrEP) 2. Magama mani lafika engcondvweni yakho nawubona letintfo leti. Kute emagama lakahle noma lakabi; Ngifuna kwati kutsi yini imicabango yakho yekucala. Yonkhe imicabango yamukelekile. 3. Kukhona yini lokutsandzako ngaloku? Ngicela ungichazele kabanti ngaloko. 4. Kukhona yini longakutsandzi ngaloku? Ngicela ungichazele kabanti ngaloku. 5. Yini umlayeto lowutfola kuloku? Ngicela ungichazele kabanti ngaloku. 6. Kukhona yini umlayeto loshodako kuloku? Yini lokunye longatsandza kukwati? 7. Nawungakhona kushintja lelipheshana noma lesitfombe noma lelikhadi, yini longakushintja nangabe kukhona? |
| 1. Ngiyabonga ngemicabango yakho ngaloku. Nyalo ngicela ucabange ngaletinye tindlela lotifundzile kuletinye tindzaba tetempilo. Ukhona yini umkhankaso wetempilo njengewe Malaria, we HIV noma we TB losemcondvweni wakho lapho weva ngatsi kukhona lokufundzako kiwo( interviewer uniketa litfuba lekutfola imphendvulo). Kuhle. Ngicela ungitjele ngawo.   Yini loyitsandzako nge (khuluma ngalomkhankaso lawushito kutsi wawutsandza).   1. Ngekubuka kwakho kukhona yini lesingakwenta lokufana naloku lokungaba kwakaPrEP? Nangabe kukhona, kungentiwa noma kwentiwe njani kuze kufanele PrEP? 2. Nangabe kute, yini leyenta ucabange kutsi PrEP akafanelwa nguloku? 3. Uye ukhulume noma ucoce ngemilayeto yetemphilo nebangani bakho noma nemndeni wakho noma nje bantfu lobatiko? 4. Ngicela ungichazele kutsi ucoca nabani? 5. Sizama kwakha imilayeto letokwatisa ngaPrEP iphindze yenta kube lula kutsi bantfu batfole PrEP. Khona lokucabangako lesingakubuka sisenta loku? |
| 1. Nyalo asesicabange ngelikusasa. Labanye bantfu nabacala iPrEP bayibuka njengeliphilisi labatalitsatsa imphilo yabo yonkhe, labanye bayibuka ngalenye indlela. 2. Utsini umuvo wakho ngekunatsa lePrEP kuletinyanga letitako? 3. Yini tintfo letingenta uchubeke utsatse iPrEP ngesikhatsi lesitako? 4. Yini locabanga kutsi kungenta kube lukhuni kutsi uhlale unatsa iPrEP? 5. Yini lengentiwa ngulabaphetse tinhlelo tetemphilo kukusita uncobe lengcinamba? |
| 1. Nawubuka emuva sikhona yini sikhatsi lofisa kube ngabe bokhona kutfola iPrEP? Ngicela ungitjele ngaloko. |
| 1. Sesigcina, yini lokubonile usatsatsa PrEP lokungentiwa ncono kuze labanye bangabi nebulukhuni noma batfole PrEP kalula. |
| 1. Sesicedza kukhona yini lengingakakubuti kona locabanga kutsi ngabe ngikubutile? |
| 1. Kukhona yini lokunye longatsandza kukungeta? |
