# Supplementary material for: “It’s hard for us men to go to the clinic. We naturally have a fear of hospitals.” Men’s risk perceptions, experiences and program preferences for PrEP: A mixed methods study in Eswatini
Source: PLoS One. 2020 Sep 23;15(9):e0237427. doi: 10.1371/journal.pone.0237427 (PMC7510987; doi:10.1371/journal.pone.0237427)
Supplement: S6 File — (DOCX) [file pone.0237427.s006.docx]

**TOOL – IN DEPTH CLIENT INTERVIEWS – PREP CONTINUED UPTAKE AND USE**

 Client experiences with use of HIV pre-exposure prophylaxis in Swaziland

Njengoba sikhulumisene masicela imvumo yakho, konkhe lotasitjela kona kutawugcinwa kuyimfihlo. Kukukhumbuta, lokucocisana kwetfu kutawutsatsa sikhatsi lesibekiselwa kumizuzu lengu 45 kuya ku 60. Ngabe kukhona yini imibuto lonayo singakacali? Ngicela kucala kutsebula ngemvumo yakho?

**__________________________________________________________________________________**

Sawubona... Siyabonga kutsi uvume kuba yincenye yalokucocisana kwetfu namuhla. Ngicele kutsi sihlangane namuhla ngenhloso yekutfola lwati lolubanti ngaloke wahlangabetana nako ngekunatsa emaphilisi ngenhloso yekuvikela kutseleleka ligciwane leHIV, lokubitwa nge pre-exposure prophylaxis (PrEP) nako konkhe lokuhambisana nelwati lwakho ngekufundza, kutfola, nekutsatsa lamaphilisi e-PrEP. Kungenteka kube nemibuto longafisi kuyiphendvula, naloko akunankinga. Khumbula kutsi awukaphoceleleki kungenela lokucocisana kwemibuto. Ngicela ukhumbule futsi kutsi kute timphendvulo letikabi. Ngifise kuva nje konkhe longakuveta noma lokucabangako.

| Question |
| --- |
| 1. Njengoba ngike ngasho phambilini, kucocisana kwetfu kutawuba nge PrEP. Ngicela ucabange ngalesikhatsi uva nge PrEP kwekucala. Usakhumbula yini ngesikhatsi uva nga PrEP kwekucala ngca? 2. Kube semcondvweni wakho kutsi kute imphendvulo lekahle noma lekabi. Ngekucondza kwakho ngabe yini PrEP? 3. Nguliphi ligama leSiSwati longalisebentisa kubita iPrEP? 4. Ngabe umuntfu angayinatselani nje iPrEP? 5. Sizatfu sini lesingenta umuntfu angayinatsi iPrEP? 6. I PrEP ilungela bani? 7. Yini lokunye lokwatiko nge PrEP? |
| 1. Nyalo ngicela ungicocele nje indzaba yakho kusukela weva nge PrEP kuze kube ngunyalo. Nangabe ute inkinga ngitocela kuhle ngikumisa kutovisisa kabanti 2. Kunini phindze kukuphi lapho weva khona nga PrEP kwekucala? 3. Wacabangani ngalesikhatsi uva ngaleliphilisi? 4. Tikhona yini letinye tintfo lowacabanga ngato nawucala kuva ngaleliphilisi? Kungaba Nguliphi lwati lolungetiwe longafisa kwati ngalo kuloku lesikhuluma ngako? 5. Yin lekwenta ucabange kutsi iPrEP iyakulungela? 6. Yin lekwenta ucabange kutsi iPrEP itakusebentela? 7. Yini lekujabulisako ngekucala iPrEP? 8. Yini lekukhatsatako ngekucala le PrEP? |
| 1. Bekukhona yini kungabata tsite ngekucala I PrEP? 2. Wabese ukhetsa njani kulokungabata kanye naletizatfu betikwenta ufune kucala I PrEP? |
| 1. Ngiyabonga kungichazela kabanti ngalondlule kiko usatsatsa PrEP. 2. Kulamaviki lambalwa utsatsa PrEP yini lowendlule kuko? 3. Yini lokuhle lokwentekile? 4. Kube yini tingcinamba? 5. Tikhona yini tingcinamba temphilo lohlangene nato? 6. Ikhona yini imivuka lebangwa ngulamaphilisi? 7. Lemivuka lebangwa ngulamaphilisi boyigadzile yini noma ikwetfusile? 8. Usebhekene nalemivuka lebangwa ngulamaphilisi, ukhona yini lebekakusita kutsi ukhone kucondzisisa? 9. Tingcinamba tini lohlangana nato ekunatseni lamaphilisi akho onkhe malanga? 10. Tintfo tini lotentile kuze ukhone kukhumbula kunatsa lamaphilisi akho? 11. Tingcinamba tiphi lohlangene nato nawuyolandza lamanye emaphilisi? 12. Taba tini letente kube melula kuphindze ubuye emtfolamphilo utolandza emaphilisi? 13. Tintfo tini letingentiwa litiko letemphilo noma tinsita letakha tinhlelo tetemphilo kutsi kube melula kubantfu labafana nawe kungenela baphindze bahlale kuleluhlelo lwe PrEP? |
| 1. Tinengi tizatfu letenta bantfu batsatse noma bangatsatsi emaphilisi abo njengoba betemphilo bakhutsata. Nasiku PrEP yini locabanga kutsi kutawenta kube matima noma kube lula kutsi wena noma labanye lobatiko batsatse I PrEP ngendlela betemphilo labakhutsata ngayo?   Ngekucabanga kwakho, yini leyenta bantfu bangafuni kungenela leluhlelo lwe PrEP ngisho nalabo labasengotini lesetulu yekutfola leligciwane?  Nawucabanga yini lengentiwa lesitako kuze kube ncono kutfola bantfu labasengotini yekutfola leligciwane kutsi bacale bangenele leluhlelo lwe PrEP? |
| 1. Nyalo ngitocela sikhulume ngetelicansi. Khumbula kutsi timphendvulo takho tiyimfihlo titawubonwa ngulabenta lucwaningo kuze kutsi kutfolakala kwe PrEP kutfutfuke. Uyeva mosi? (Pause). Ngicela sikhulume ngetelicansi kanye ne PrEP (pause). 2. Ucabanga kutsi I PrEP ingayitsikabeta kanjani imphilo yakho yetelicansi? 3. Uke wacoca yini nesingani sakho mayelana ne PrEP. 4. Uma ukile wacoca naye, yahamba njani lenkhulumo yenu? |
| 1. Lyitsikabetile yini iPrEP imphiloyakho yasecansini? 2. Kukutsikabetile yini kutsi ungacoca njani ngesimo sakho semphilo kulabo lotsandzana nabo? 3. Sibekhona yini sitsikabeto tsite ekusebentiseni kwakho lijazi lemkhwenyane? 4. Kusifakile yini sandla kubantfu lolalana nabo? |
| 1. Kuyintfo leyindzabamlonyeni yini le PrEP eveni laKaNgwane kutsi bangani netingani tingakhona kucoca ngayo ngalokusebaleni? 2. Uma kwenteka, kwentiwa yini futsi uma kungenteki, kubangelwa yini? |
| 1. Ukhona yini lomatiko lonatsa I PrEP? Imivo yabo yekucala PrEP iyafana yini noma ihlukile kunaleyakho? 2. Nacala njani kucoca nge PrEP nalona muntfu? |
| 1. Cabanga umngani lomatiko loku PrEP, ucabanga kutsi ngabe yini tinkinga labadibana nabo nabazama kunatsa I PrEP? 2. Usho kutsi lenkinga yakhe ihlupha bonkhe bantfu noma umngani wakho kuphela? |
| 1. Nawucabanga ngelive laKaNgwane noma ngemmango wangakini, tintfo tini letingenta kube lukhuni kutsi bantfu labafana nawe bakhone kungenela loluhlelo lwe PrEP. Tingashintjwa njani leto tintfo kuze kutsi kube melula kutsi bantfu KaNgwane labafana nawe bakhone kungenela loluhlelo lwe PrEP? |
| 1. Nyalo ngitawutsandza kukukhombisa natintfo letiphatselene naPrEP lokungenteka kutsi ukewakubona phambilini noma awukake ukubone. (lobutako atjengise lophendvulako letintfo takaPrEP)   Magama mani lafika engcondvweni yakho nawubona le flyer noma le poster lena. Kute emagama lakahle noma lakabi; Ngifuna kwati kutsi yini imicabango yakho yekucala. Yonkhe imicabango yamukelekile.   1. Kukhona yini lokutsandzako ngaloku? Ngicela ungichazele kabanti ngaloko. 2. Kukhona yini longakutsandzi ngaloku? Ngicela ungitjele kabanti ngaloko 3. Yini umlayeto lowutfola kuloku? Ngicela ungichazele kabanti ngaloko. 4. Kukhona yini umlayeto loshodako kuloku? Yini lokunye longatsandza kukwati? 5. Nawungakhona kushintja lelipheshana noma lesitfombe noma lelikhadi, yini longakushintja nangabe kukhona? |
| 1. Ngiyabonga ngemicabango yakho ngaloku. Nyalo ngicela ucabange ngaletinye tindlela lotifundzile kuletinye tindzaba tetempilo. Ukhona yini umkhankaso wetempilo njengewe Malaria, we HIV noma we TB losemcondvweni wakho lapho weva ngatsi kukhona lokufundzako kiwo( interviewer uniketa litfuba lekutfola imphendvulo). Kuhle. Ngicela ungitjele ngawo.    Yini loyitsandzako nge (khuluma ngalomkhankaso lawushito kutsi wawutsandza).   1. Ngekubuka kwakho kukhona yini lesingakwenta lokufana naloku lokungaba kwakaPrEP? Nangabe kukhona, kungentiwa noma kwentiwe njani kuze kufanele PrEP? Nangabe kute, yini leyenta ucabange kutsi PrEP akafanelwa nguloku? 2. Uye ukhulume noma ucoce ngemilayeto yetemphilo nebangani bakho noma nemndeni wakho noma nje bantfu lobatiko? Ngicela ungichazele kutsi ucoca nabani? 3. Sizama kwakha imilayeto letokwatisa ngaPrEP iphindze yenta kube lula kutsi bantfu batfole PrEP. Khona lokucabangako lesingakubuka sisenta loku? |
| 1. Nyalo asesicabange ngelikusasa. Labanye bantfu nabacala iPrEP bayibuka njengeliphilisi labatalitsatsa imphilo yabo yonkhe, labanye bayibuka ngalenye indlela. 2. Utsini umuvo wakho ngekunatsa lePrEP kuletinyanga letitako? 3. Yini tintfo letingenta uchubeke utsatse iPrEP ngesikhatsi lesitako? 4. Yini locabanga kutsi kungenta kube lukhuni kutsi uhlale unatsa iPrEP? 5. Yini lengentiwa ngulabaphetse tinhlelo tetemphilo kukusita uncobe lengcinamba? |
| 1. Nawubuka emuva sikhona yini sikhatsi lofisa kube ngabe bokhona kutfola iPrEP? 2. Ngicela ungitjele ngaloko.? |
| 1. Sesigcina, yini lokubonile usatsatsa PrEP lokungentiwa ncono kuze labanye bangabi nebulukhuni noma batfole PrEP kalula? |
| 1. Sesicedza kukhona yini lengingakakubuti kona locabanga kutsi ngabe ngikubutile? |
| 1. Kukhona yini lokunye longatsandza kukungeta? |
