# Supplementary material for: “It’s hard for us men to go to the clinic. We naturally have a fear of hospitals.” Men’s risk perceptions, experiences and program preferences for PrEP: A mixed methods study in Eswatini
Source: PLoS One. 2020 Sep 23;15(9):e0237427. doi: 10.1371/journal.pone.0237427 (PMC7510987; doi:10.1371/journal.pone.0237427)
Supplement: S7 File — (DOCX) [file pone.0237427.s007.docx]

**QUALITATIVE TOOL – IN DEPTH CLIENT INTERVIEWS – PREP DECLINE**

**Client motivations to decline PrEP offer**

As we went over in the consent, all of the information you provide will be kept confidential. Just as a reminder our interview will probably last around 45-60 minutes. Do you have any questions before we begin? May I start the recording? *[Start recording]*

**Good [afternoon/morning] thank you for participating today**! I have asked you to meet with me in the hopes of learning more about your experience with HIV prevention services offered in this facility and more specific about pre-exposure prophylaxis (PrEP). Some of the questions I will ask, you may not want to answer and that is fine. Remember that your answers are confidential and participation is completely voluntary. Also please keep in mind that there are no right or wrong answers, I am interested in anything you can share with me.

Questions for participants who did not initiate PrEP

| **Question** |
| --- |
| 1. As I mentioned earlier the main focus of our discussion is PrEP. Please think of the first time you heard about PrEP. Do you remember the first time you heard about it? (Pause, await respondent) Okay, good.   Keeping in mind that there are no right or wrong answers, in your understanding,   1. What is PrEP? 2. What would be a name that you would give PrEP? 3. Why would a person take PrEP? 4. Why would a person not take PrEP? 5. Who is PrEP for? 6. What else do you know about PrEP? |
| Now please walk me through your story from when you heard about PrEP until now. If you don’t mind, I’ll interrupt sometimes to get more details.  When and where did you first hear about PrEP  What did you think when you heard about this pill?  Were there any things that you wondered about when you first heard about this pill?  If you could have had more information, what would you have liked to know?   - 1. What made you think that PrEP did not make sense for you?   2. Were there any things that made you worried or concerned about starting PrEP? |
| 1. Let us imagine that there is a woman. She does not know her husband’s HIV status. She thinks he may have other sexual partners.    1. Would it make sense for her to take PrEP?    2. What would be some of the benefits for a person like her taking PrEP?    3. What would be some of the drawbacks? |
| 1. Let us imagine that there is a man. He knows his wife is HIV positive. He is negative.    1. Would it make sense for him to take PrEP?    2. What would be some of the benefits for a person like him taking PrEP?    3. What would be some of the drawbacks of him taking PrEP? |
| 1. Is PrEP a topic that men and women in Swaziland feel comfortable discussing with friends and/or partners?    1. Why or why not? |
| 1. There are a lot of reasons that people take or don’t take medicine even if providers would like people to take a medicine. The Ministry of Health wants to learn how to make more Swazis interested in taking PrEP. The people in the Ministry can’t talk to everyone in Swaziland, so the thoughts you can share are very valuable to me. Your opinion represents many many people who we cannot talk to directly.    1. In your opinion, why do you think some people – even those who are at risk for HIV – do not want to take PrEP?    2. Changing perceptions of what it means to live with HIV?    3. Changing perception of what it means to be “at risk” for HIV?    4. How could the Ministry get more people interested in PrEP?    5. How could things be changed to make it easier for you and others in Swaziland to get and routinely take PrEP? |
| 1. Do you know anyone else who is currently taking PrEP? Have you ever discussed PrEP with another person? How did the conversation go? |
| 1. Now I would like to show you some materials about PrEP that you might or might not have seen before. (Interviewer shows the standard MoH PrEP material to the respondent)   Please tell me some words that come to your mind when you see this flyer/poster. There are no right or wrong words; I am looking to learn from your first impressions and thoughts. All thoughts are welcome.   - - 1. PROBES- Is there anything you like about this? Please tell me more about that.     2. PROBES- Is there anything you don’t like about this. Please tell me more about that.     3. PROBES- What do you read as the main message from this? Please tell me more about that.     4. PROBES- What message is missing from this flyer? What more would you like to know?     5. PROBES- If you would be able to change this flyer/ poster/palm card, what would you change (if at all)? |
| 1. Thank you for your thoughts about this. Now I would like you to think about other ways that you have learned about other health matters. Do you have a health campaign like for malaria or HIV or TB in mind where you felt like you learned something (interviewer pauses for confirmation)? Good. Now please tell me about that.    1. PROBES- What did you like about (INSERT RESPONDENTS FAVORITE MESSAGING MECHANISM)    2. PROBES- Do you think we could do something similar for PrEP messaging. If yes, how would/ should it be tailered to PrEP. If not, why do you think PrEP doesn’t fit with (it)?    3. PROBES- Do you talk about health messages with your friends, family or others you know? Please explain with who?    4. PROBES- We are trying to make the most effective, informative PrEP messaging possible and to make it easier for people to access PrEP. Can you think of anything else that we should consider in order to make it possible? |
| 1. Is there a time earlier in life when, looking back now, you wish you would have had access to PrEP? Please tell me about it. |
| 1. Can you imagine a time in the coming months or years when you might be willing to come back to learn more about PrEP or to request PrEP medicines? |
| 1. To conclude, what would be your recommendation to improve your experience with PrEP? |
| 1. What would you recommend to improve others’ experience and access to PrEP? |
| 1. And finally, is there anything that I didn’t ask you that I should have asked you? |
| 1. Is there anything else that you would like to add? |

We have come to the conclusion of the topics I had prepared to discuss today. Are there any further comments you would like to add?

**THANK YOU FOR YOUR TIME!**
