# Supplementary material for: “It’s hard for us men to go to the clinic. We naturally have a fear of hospitals.” Men’s risk perceptions, experiences and program preferences for PrEP: A mixed methods study in Eswatini
Source: PLoS One. 2020 Sep 23;15(9):e0237427. doi: 10.1371/journal.pone.0237427 (PMC7510987; doi:10.1371/journal.pone.0237427)
Supplement: S8 File — (DOCX) [file pone.0237427.s008.docx]

**QUALITATIVE TOOL – IN DEPTH CLIENT INTERVIEWS – PREP DECLINE**

Client motivations to decline PrEP offer

Njengoba sikhulumisene masicela imvumo yakho, konkhe lotasitjela kona kutawugcinwa kuyimfihlo. Kukukhumbuta, lokucocisana kwetfu kutawutsatsa sikhatsi lesibekiselwa kumizuzu lengu 45 kuya ku 60. Ngabe kukhona yini imibuto lonayo singakacali? Ngicela kucala kutsebula ngemvumo yakho?

**__________________________________________________________________________________**

 Sawubona... Siyabonga kutsi uvume kuba yincenye yalokucocisana kwetfu namuhla. Ngicele kutsi sihlangane namuhla ngenhloso yekutfola lwati lolubanti ngaloke wahlangabetana nako ngekunatsa emaphilisi ngenhloso yekuvikela kutseleleka ligciwane leHIV, lokubitwa nge pre-exposure prophylaxis (PrEP) nako konkhe lokuhambisana nelwati lwakho ngekufundza, kutfola, nekutsatsa lamaphilisi e-PrEP. Kungenteka kube nemibuto longafisi kuyiphendvula, naloko akunankinga. Khumbula kutsi awukaphoceleleki kungenela lokucocisana kwemibuto. Ngicela ukhumbule futsi kutsi kute timphendvulo letikahle noma letingasiko kahle. Ngifise kuva nje konkhe longakuveta noma lokucabangako.

Questions for participants who did not initiate PrEP

| Question |
| --- |
| 1. Njengoba sike sakhuluma phambilini, kucocisana kwetfu kutawuba nge PrEP. Ngicela ucabange ngalesikhatsi uva nge PrEP kwekucala. Usakhumbula yini ngesikhatsi uva nga PrEP kwekucala ngca? 2. Kube semcondvweni wakho kutsi kute imphendvulo lekahle noma lekabi. Ngekucondza kwakho ngabe yini PrEP? 3. Yini ligama leSiswati longalinika  PrEP? 4. Ngabe umuntfu angayinatselani nje I PrEP? 5. Ngabe umuntfu angete ayitsatselani I PrEP? 6. I PrEP ilungela bani? 7. Yini lokunye lokwatiko nge PrEP? |
| 1. Nyalo ngicela ungicocele nje indzaba yakho kusukela weva nge PrEP kuze kube ngunyalo. Nangabe ute inkinga ngitocela kuhle ngikumisa kutovisisa kabanti 2. Kunini phindze kukuphi lapho weva khona nga PrEP kwekucala? 3. Wacabangani nawuva ngePrEP? 4. Ngabe kukhona yini tintfo lowaticabanga nawucala kuva ngaleliphilisi? Nangabe bowungaba nalo lolunye lwati yini longatsandza ku  kwati nyalo? 5. Yini leyakwenta ucabange kutsi  PrEP ayikulungeli? 6. Ngabe kukhona tintfo letakwenta wakhatsateka ngekucala PrEP? |
| 1. Ase sibekise kutsi kunaMake,akasati simo sengati semyeni wakhe ucabanga kutsi unalabanye laya nabo ecansini? 2. Kungaba ngumcondvo lomuhle yini kutsi anatse iPrEP? 3. Kungaba yini tinzuzo temuntfu lofana naye kutsi anatse PrEP? 4. Yini lengenta angawanatsi? |
| 1. Ase sibekise kutsi kuna Babe, uyati kutsi Make wakakhe uphila neligciwane le HIV yena ute leligciwane 2. Kungaba ngumcodvo lomuhle yini kutsi anatse iPrEP? 3. Kungaba yini tinzuzo kumuntfu lofana  naye ngekunatsa  iPrEP? 4. Yini tintfo letingenta angawanatsi lamaphilisi ePrEP? |
| 1. IPreP iyintfo leyindzabamlonyeni yini eveni lakaNgwane kutsi boMake nabo Babe netingani tingakhona kucoca ngayo ngalokusebaleni? Uma kwenteka, kwentiwa yini futsi uma kungenteki kubangelwa yini? |
| 1. Tinyenti tizatfu letenta bantfu balinatse noma bangalinatsi leliphilisi noma ngabe  tisebenti tetemphilo tingatsandza kutsi bantfu balinatse . Litiko letemphilo lifuna kufundza kabanti kutsi lingentanjani kwenta emaSwati abe nemndladla wekungenela iPrEP, ngako-ke umbono wakho umcoka kimi futsi umelela bantfu labanyenti lesingeke sikhone kukhuluma nabo ngco. 2. Ngembono wakho ucabanga kutsi yini bantfu banganatsi emaphilisi ePrEP, kanye nalabo labasengotini ye HIV? 3. Kushintja imibono ngekutsi kusho kutsini kuphila neligciwane le HIV ? 4. Imibono ngekutsi kusho kutsini kubasengotini yekutfola iHIV? 5. Yini lengentiwa litiko letemphilo kuze bantfu babe nenshisekelo nge PrEP? 6. Yini tintfo letingentiwa kuze kutsi kube lula kuwe nalabanye kangwane  batfole phindze banatse iPrEP ngendlela lefanele |
| 1. Ukhona yini lomatiko lonatsa I PrEP?kuke kwenteka wacocisana nalomunye nge PrEP?Yahamba njani lengcogciswano yenu |
| 1. Nyalo ngitawutsandza kukukhombisa natintfo letiphatselene naPrEP lokungenteka kutsi ukewakubona phambilini noma awukake ukubone. (lobutako atjengise lophendvulako letintfo takaPrEP) 2. Magama mani lafika engcondvweni yakho nawubona letintfo leti. Kute emagama lakahle noma lakabi; Ngifuna kwati kutsi yini imicabango yakho yekucala. Yonkhe imicabango yamukelekile. 3. Kukhona yini lokutsandzako ngaloku? Ngicela ungichazele kabanti ngaloko. 4. Kukhona yini longakutsandzi ngaloku? Ngicela ungichazele kabanti ngaloku 5. Yini umlayeto lowutfola kuloku? Ngicela ungichazele kabanti ngaloku 6. Kukhona yini umlayeto loshodako kuloku? Yini lokunye longatsandza kukwati? 7. Nawungakhona kushintja lelipheshana noma lesitfombe noma lelikhadi, yini longakushintja nangabe kukhona? |
| 1. Ngiyabonga ngemicabango yakho ngaloku. Nyalo ngicela ucabange ngaletinye tindlela lotifundzile kuletinye tindzaba tetempilo. Ukhona yini umkhankaso wetempilo njengewe Malaria, we HIV noma we TB losemcondvweni wakho lapho weva ngatsi kukhona lokufundzako kiwo( interviewer uniketa litfuba lekutfola imphendvulo). Kuhle. Ngicela ungitjele ngawo.   Yini loyitsandzako nge (khuluma ngalomkhankaso lawushito kutsi wawutsandza).   1. Ngekubuka kwakho kukhona yini lesingakwenta lokufana naloku lokungaba kwakaPrEP? Nangabe kukhona, kungentiwa noma kwentiwe njani kuze kufanele PrEP? Nangabe kute, yini leyenta ucabange kutsi PrEP akafanelwa nguloku? 2. Uye ukhulume noma ucoce ngemilayeto yetemphilo nebangani bakho noma nemndeni wakho noma nje bantfu lobatiko? Ngicela ungichazele kutsi ucoca nabani? 3. Sizama kwakha imilayeto letokwatisa ngaPrEP iphindze yenta kube lula kutsi bantfu batfole PrEP. Khona lokucabangako lesingakubuka sisenta loku? |
| 1. Nawubuka emuva sikhona yini sikhatsi lofisa kube ngabe bokhona kutfola iPrEP? Ngicela ungitjele ngaloko. |
| 1. Nawucabanga kulesikhatsi lesitako utawufuna kubuya utofundza kabanti nge PrEP noma utocela liphilisi le PrEP? |
| 1. Sesigcina, yini lokubonile usatsatsa PrEP lokungentiwa ncono kuze labanye bangabi nebulukhuni noma batfole PrEP kalula? |
| 1. Sesicedza kukhona yini lengingakakubuti kona locabanga kutsi ngabe ngikubutile? |
| 1. Kukhona yini lokunye longatsandza kukungeta? |

We have come to the conclusion of the topics I had prepared to discuss today. Are there any further comments you would like to add?

THANK YOU FOR YOUR TIME!
