# Supplementary material for: “It’s hard for us men to go to the clinic. We naturally have a fear of hospitals.” Men’s risk perceptions, experiences and program preferences for PrEP: A mixed methods study in Eswatini
Source: PLoS One. 2020 Sep 23;15(9):e0237427. doi: 10.1371/journal.pone.0237427 (PMC7510987; doi:10.1371/journal.pone.0237427)
Supplement: S9 File — (DOCX) [file pone.0237427.s009.docx]

**QUALITATIVE TOOL – HEALTH WORKER INTERVIEWS**

**Health care worker’s experiences with HIV pre-exposure prophylaxis in Swaziland**

As we went over in the consent, all of the information you provide will be kept confidential. Just as a reminder our interview will probably last around 45 minutes. Do you have any questions before we begin?  May I start the recording? *[Start recording]*

**Good [afternoon/morning], thank you for participating today**! I have asked you to meet with me in the  hopes of learning more about your experience providing pre-exposure prophylaxis (PrEP). Remember  that your answers are confidential and participation is completely voluntary. Also please keep in mind that there are no right or wrong answers, I am interested in anything you can share with me.

| **Questions for healthcare workers involved in PrEP provision** |
| --- |
| 1. As I mentioned earlier, the main focus of our discussion is PrEP. 2. Have you heard of PrEP? 3. Where did you first hear about PrEP? 4. What have you heard about PrEP? 5. How do providers talk about PrEP with one another? |
| 1. Please describe the training you received on PrEP. 2. Did you complete the training? What were the trainings’ strengths and weaknesses? 3. Did the training adequately prepare you to administer and counsel clients on PrEP? 4. What could have made the training better? |
| 1. HIV has been around a long time. And in the past decades, a lot of interventions and programs to address HIV have been introduced in Swaziland. How do you feel about PrEP as an additional HIV prevention strategy? |
| 1. Now I want to talk about offering PrEP to patients. 2. According to what you were told, who is PrEP “for,” by this I mean, for whom does PrEP make sense? 3. The people who design PrEP programs are often not in Swaziland. They sit in far away offices, and don’t really know what its like to work in a health facility in Swaziland. So what is your personal opinion about who should be offered PrEP? 4. What are some of the things that you have to think about as a provider in this facility whether to offer PrEP? 5. What are some of the things that you have to think about as a provider in this facility when to offer PrEP? 6. What are some of the things that you have to think about as a provider in this facility how to offer PrEP? 7. What are some of the things that you have to think about as a provider in this facility to whom to offer PrEP? 8. Has there been a time when you hesitated or decided against offering PrEP? 9. Has there been a time when you noticed that colleagues hesitated or decided against offering PrEP? |
| 1. Now I want to talk about not offering PrEP to clients. 2. According to what you were told, who is PrEP not “for,” by this I mean, for whom does PrEP not make sense? 3. Are there clients who should not be given PrEP when they request it? |
| 1. In similar settings both within and outside Swaziland (upon the introduction of other HIV-related treatment) we have noticed that providers and clients are hesitant to offer PrEP. 2. Has there been a time when you hesitated or decided against offering PrEP? 3. Has there been a time when you noticed that colleagues hesitated or decided against offering PrEP? |
| 1. How has offering PrEP services affected your day-to-day workload in the facility, if at all? |
| 1. Is there anything you feel you need (that you currently do not have) that could support you to adequately implement PrEP at your facility? |
| 1. How have clients responded when you recommend that they use PrEP? 2. Have clients that you have interacted with had a positive experience with it? 3. What benefits and challenges did they highlight? |
| 1. Now I would like to talk about the PrEP Information, education and communication (IEC) material that is available in your facility.  (Interviewer shows the material to the respondent)   Please tell me some words that come to your mind when you see this flyer/poster. There are no right or wrong words; I am looking to learn from your first impressions and thoughts. All thoughts are welcome.   1. Is there anything you like about this? Please tell me more about that. 2. Is there anything you don’t like about this. Please tell me more about that. 3. What do you read as the main message from this? Please tell me more about that. 4. If you would be able to change the message/ content, is there anything you would like to change? Please explain. |
| 1. Thank you for your thoughts about the IEC material. Now I would like you to think about other ways that you know to promote health services or to educate clients about health services. Please let us pause and think of another health campaign that you think worked well. 2. Why do you think that campaign worked well? 3. Do you think we could do something similar for PrEP messaging. If yes, how would/ should it be tailored to PrEP? If not, why do you think PrEP doesn’t fit with that (FAVORED MESSAGING MECHANISM) 4. Can you think of a time when a client referred to a particular HIV prevention program as their reason for using that prevention method? If yes, do you know which one it was? 5. We are trying to make the most effective, informative PrEP messaging possible and to make it easier for providers to offer PrEP and people to access PrEP. Can you think of anything else that we should consider in order to make it possible? |
| 1. Do you have any suggestion on how to improve PrEP uptake in your facility? |
| 1. If you were sitting with the Ministry and you could tell them just a few things they should think about in terms of challenges you face to offering PrEP, what would be the main things you would want the Ministry to know? |
| 1. Is there anything I didn’t ask you that I should have asked you in relation to PrEP? |
| 1. Is there anything else you would like to tell me |

We have come to the conclusion of the topics I had prepared to discuss today. Are there any further comments you would like to add? **THANK YOU FOR YOUR TIME!**
