# Supplementary material for: “It’s hard for us men to go to the clinic. We naturally have a fear of hospitals.” Men’s risk perceptions, experiences and program preferences for PrEP: A mixed methods study in Eswatini
Source: PLoS One. 2020 Sep 23;15(9):e0237427. doi: 10.1371/journal.pone.0237427 (PMC7510987; doi:10.1371/journal.pone.0237427)
Supplement: S10 File — (DOCX) [file pone.0237427.s010.docx]

**QUALITATIVE TOOL – HEALTH WORKER INTERVIEWS**

Health care worker’s experiences with HIV pre-exposure prophylaxis in Swaziland

Njengoba sikhulumisene masicela imvumo yakho, konkhe lotasitjela kona kutawugcinwa kuyimfihlo. Kukukhumbuta, lokucocisana kwetfu kutawutsatsa sikhatsi lesibekiselwa kumizuzu lengu 45 kuya ku 60. Ngabe kukhona yini imibuto lonayo singakacali? Ngicela kucala kurekhoda ngemvumo yakho?

 Sawubona, ngicele kutsi uhlangane nami ngesizatfu sekutsi ngifundze kabanti ngekuniketa ipre-exposure prophylaxis (PrEP). Khumbula kutsi konkhe lotokusho kutohlala kuyimfihlo futsi awukaphoceleleki kuphendvula. Akube semcondvweni wakho kutsi kute timphendvulo letikabi. Ngiyakukhutsalela lofise kutsi ngikwati.

| Questions for healthcare workers involved in PrEP provision |
| --- |
| 1. Njengoba ngingishito phambilini, ingcikitsi yenkhulumo yetfu ngu PrEP. 2. Kukhona yini lapho uve khona ngePrEP? 3. Ngukuphi lapho ucale khona kuva nga PrEP? 4. Yini lokuvile nga PrEP? 5. Titsini letinye tisebenti tetemphilo naticoca ngePrEP? |
| 1. Ngicela ungichazele ngetinfundziso lotitfolile ngePrEP? 2. Ngabe wakhona yini kutihambela uticedze tonkhe letifundziso? 3. Ngabe yini lokwahamba kahle nalokwahamba kabi kuletimfundziso? 4. Ngabe letimfundziso takuhlomisa yini kutsi ukhone kutilungiselela nekululeka labafunalusito ngePrEP? 5. Ngabe yini lobekungenta kutsi letimfundziso tibe ncono? |
| 1. HIV sifo lesesinesikhatsi sikhona. Phambilini, minyenti imetamo netinhlelo letibukete iHIV letaletfwa kaNgwane. Itsini imivo yakho ngePrEP njengoba yengetwa njengendlela yekuvikela iHIV? |
| 1. Nyalo ngifuna sibuke kuniketa PrEP kulabanyidzingako. 2. Nasibuka lotjelwe kona, inikwa bobani iPrEP, ngaloku ngitsi bobani lova ngatsi balungelwa nguPrEP? 3. Ngemivo yakho noma losokubonile noma londlule kiko, iPrEP ilungela bani, ngaloko ngitsi kukhona lokubhaliwe ngekutsi yabobani, kepha ngalokunye tikhona tingcinamba tasesisesibhedlela lokungaba lukhuni kubantfu labakha loluhlelo labangekho kulendzawo noma labahleti emahhovisi labangeke bakucondze. Ngekucabanga kwakho, iPrEP ilungela bobani? 4. Ucabanga kutsi bobani labangazuza kakhulu nge PrEP? |
| 1. Nyalo ngifuna kukhuluma ngekunganiketi iPrEP kulabeta esibhedlela. 2. Kulofundziswe kona, bobani labangalungeli kutfola PrEP, ngaloku ngitsi iPrEP bobani lengabalungeli? 3. Bakhona yini labeta batofuna PrEP lokumele banganikwa nabayifuna? |
| 1. Etindzaweni letifana naleti kungaba kaNgwane noma ngephandle (nakucalwa letinye tinhlelo letihambelana nekunaka ehlangotsini leHIV) sibonile betemphilo nalabeta esibhedlela baba nekungabata kuniketa iPrEP. 2. Ngabe sikhona yini sikhatsi lapho khona ube nekungabata noma ube nemuvo lowehlukile ekunikeni PrEP? 3. Ngabe sikhona yini sikhatsi lapho khona balingani bakho babe nekungabata noma babe nekuphikisana ekunikeni PrEP? |
| 1. Ngabe PrEP kukhona sandla lokusifakile enanini lemsebenti wakho lapho esibhedlela? |
| 1. Ngabe kukhona yini lova ngatsi uyakudzinga (lote kona) lokungakusita kabanti kutsi ukhone kuchuba luhlelo lwaPrEP esibhedlela? |
| 1. Ngabe labeta esibhedlela bakutsatsa njani nawuncoma kutsi bangakhona kusebentisa PrEP? 2. Ngabe lococisene nabo bamtsetse kahle kangakanani? Yini tinzuzo netingcinamba labatibekile? |
| 1. Nyalo ngitotsandza kutsi sibuke ema IEC material letikhona emtfolamphilo wakho.    Yini lokufika engcondvweni nawubona le-flyer nome iposter. Kute emagama lakahle noma lakabi; Ngifuna kufundza kabanti ngemicabango nemizindlo yakho. Konkhe lokucabangako kwemukelekile.   1. Kukhona yini lokutsandzako ngaloku? Ngicela ungcitjele kabanti ngako. 2. Kukhona yini longakutsandzi ngaloku? Ngicela ungitjele kabanti ngako. 3. Ngabe yini umlayeto lomkhulu lowutfola kuloku? Ngicela ungcitjele kabanti ngako. 4. Nakungatsiwa khona longakushintja kulomlayeto, kukhona yini longafisa kukushintja. Ngicela ungichazele. |
| 1. Ngiyabonga imivo yakho ngale IEC material. Nyalo ngicela ucabange lokunye lokungasita kwatisa tetemphilo noma kufundzisa ngetemphilo kulabete esibhedlela ngetemphilo. Asesitsatse umzuzu sicabange ngalomunye umkhankhaso wetemphilo locabanga kutsi wasebenta kahle kakhulu. 2. Yini ucabange kutsi lomkhankhaso usebente kahle kakhulu? 3. Ucabanga kutsi kukhona yini lesingakwenta lokufananako ekwatiseni ngaPrEP. Nangabe yebo, yini lengenteka noma lengentiwa kutsi kuhambisane nePrEP? Nangabe cha, Yini lokwenta ucabange kutsi loko ngeke kuhlangane ne PrEP. ( Umkhankaso lowutsandzako) 4. Ngabe sikhona yini sikhatsi lapho khona lote esibhedlela akhulume ngeluhlelo lwekuvikela iHIV , lalufunako netizatfu tekusebentisa lelohlelo. Nangabe yebo, ngabe uyalwati yini lolohlelo? 5. Sizama kwenta umlayeto lonelwati, loyimphumelelo we PrEP kwenta kube ncono kubantfu kutsi bangenele PrEP. Ngicela ucabange lokunye lesingakubuka kwenta lomlayeto uphumelele? |
| 1. Unawo yini umbono longenta kutsi bantfu bangenele loluhlelo lwaka PrEP kulomtfolamphilo? |
| 1. Kube uhleti nebaphatsi betemphilo, kunetintfo lobofise kubatjela kona ngetingcinambha lenihlangabetana nato nisaniketa PrEP, yini kakhulu kati longafisa kubatjela kona? |
| 1. Ngabe kukhona yini lengingakakubuti kona lekumele ngabe ngikubutile mayelana nePrEP? |
| 1. Kukhona yini lokunye longatsandza  kukwengeta? |

We have come to the conclusion of the topics I had prepared to discuss today. Are there any further comments you would like to add? THANK YOU FOR YOUR TIME!
