# Supplementary material for: “It’s hard for us men to go to the clinic. We naturally have a fear of hospitals.” Men’s risk perceptions, experiences and program preferences for PrEP: A mixed methods study in Eswatini
Source: PLoS One. 2020 Sep 23;15(9):e0237427. doi: 10.1371/journal.pone.0237427 (PMC7510987; doi:10.1371/journal.pone.0237427)
Supplement: S11 File — (DOCX) [file pone.0237427.s011.docx]

QUALITATIVE TOOL – PREP STAKEHOLDERS INTERVIEWS

**PrEP stakeholders in Swaziland**

As we went over in the consent, all of the information you provide will be kept confidential. Just as a reminder our interview will probably last around 45-60 minutes. Do you have any questions before we begin? May I start the recording? *[Start recording]*

**Good [afternoon/morning] thank you for participating today**! I have asked you to meet with me in the hopes of learning more about your experience with health education, information and communication material related to pre-exposure prophylaxis (PrEP), and to learn your thoughts about PrEP in general. As an intervention for the PrEP demonstration project in Hhohho region, we are trying to have some initial information gathering on the design for the most feasible, acceptable and sustainable PrEP Promotion Package. Some of the questions I will ask you may not want to answer and that is fine. Remember that your answers are confidential and participation is completely voluntary. Also please keep in mind that there are no right or wrong answers, I am interested in anything you can share with me.

Questions for PrEP stakeholders:

| **Question** |
| --- |
| 1. To begin, I was hoping you could tell me a bit more about yourself?    1. Can you tell me a bit more about your position and responsibilities?    2. How do you fit within the PrEP “world”? |
| Can you tell me where you first heard about PrEP?  What were your very first thoughts upon hearing about this prevention regimen? |
| Sadly, HIV has been around a long time. And in recent decades, a lot of interventions and programs to address HIV have been introduced in Swaziland. How do you feel about PrEP as an additional HIV prevention strategy?  What are some things about PrEP that make you feel hopeful?  What are some things about PrEP that make you feel skeptical? |
| Now I would like to show you some of the information, education and communication material that are available at select facilities in relation to PrEP.  Have you seen this before?  Please tell me some words that come to your mind when you see this flyer/poster. There are no right or wrong words.  Is there anything you like about this? Please tell me more about that.  Is there anything you don’t like about this. Please tell me more about that.  If you could change the message/ content, is there anything you would like to change? What would you add? What would you remove? Please explain. |
| From your experience, which methods have you seen working in other health related interventions in the past? When I say “working,” I mean that the interventions compelled patients to seek the care that the message intended.  What do you think it was about those interventions that made them work well?  How could we adapt the effective ingredients of that intervention for PrEP? Please provide as much detail as possible. |
| Thanks for this lively conversation. I want to now talk about some bigger picture issues in relation to PrEP. By bigger picture, I mean some of the larger forces that can affect the day-to-day routines of getting a treatment regimen like PrEP introduced as part and parcel of care provided in faciltiies. Please tell me about some of the “bigger forces” that have affected the introduction and uptake of PrEP.  Probe on novelty or newness of PrEP  Probe on PrEP and issues of morality/ social mores/ social pressure  Probe on PrEP and risk compensation  Probe on economic situation in country  Probe on political situation in country  What do you feel is needed in order to get PrEP adopted in Swaziland as a National policy? |
| 1. We are nearing the end. Now, I'd like to think about the future in relation to PrEP. What do you envision as the future of PrEP?    1. If we assume for a moment that PrEP would continue what are some of your hopes, plans, concerns in relation to PrEP? |
| We are trying to make the most effective, informative PrEP messaging possible and to make it easier for people to access PrEP. Can you think of anything else that we should consider (whether a big issue in relation to PrEP as a topic or a specific suggestion in relation to the IEC materials) in order to make PrEP accessible, affordable and available to people in Swaziland? |
| 1. Is there anything I haven’t asked you that I should have asked you? |

We have come to the conclusion of the topics I had prepared to discuss today. Are there any further comments you would like to add? **THANK YOU FOR YOUR TIME!**
