# Supplementary material for: “It’s hard for us men to go to the clinic. We naturally have a fear of hospitals.” Men’s risk perceptions, experiences and program preferences for PrEP: A mixed methods study in Eswatini
Source: PLoS One. 2020 Sep 23;15(9):e0237427. doi: 10.1371/journal.pone.0237427 (PMC7510987; doi:10.1371/journal.pone.0237427)
Supplement: S12 File — (DOCX) [file pone.0237427.s012.docx]

QUALITATIVE TOOL – COMMUNITY LEADER INTERVIEWS

**Community leaders in Swaziland**

As we went over in the consent, all of the information you provide will be kept confidential. Just as a reminder our interview will probably last around 45-60 minutes. Do you have any questions before we begin? May I start the recording? *[Start recording]*

**Good [afternoon/morning] thank you for participating today**! I have asked you to meet with me in the hopes of learning more about your experience and perceptions related to a new HIV prevention strategy called pre-exposure prophylaxis (PrEP), You have been identified by men and women in your community as a key figure and community leader. We are interested to learn your thoughts regarding PrEP because you may have some influence regarding how PrEP is seen within your community. Some of the questions I will ask you may not want to answer and that is fine. Remember that your answers are confidential and participation is completely voluntary. Also please keep in mind that there are no right or wrong answers, I am interested in anything you can share with me.

Questions for PrEP community leaders:

| **Question** |
| --- |
| 1. To begin, I was hoping you could tell me a bit more about yourself?    1. Can you tell me a bit more about your position and responsibilities?   How do you represent your community?   - 1. Please give me an example of a health matter you have discussed with a community member or the community generally. |
| 1. Can you talk me through how you have spoken with your community regarding HIV prevention strategies? |
| Please tell me about what makes advising your community regarding matters relating to HIV and sex difficult.  Probe on culture  Probe on religion  Probe on gender  Probe on age  Probe on knowledge levels  Probe on stigma |
| Please tell me about what makes advising your community regarding matters relating to HIV and sex easy. |
| What have you heard about PrEP? |
| How do you feel about PrEP as an HIV prevention method? |
| Who do you think PrEP should be for? |
| Who do you think PrEP shouldn’t be for? |
| Can you tell me what you think PrEP means for your community? |
| Can you tell me about who you think will benefit the most from PrEP in your community? |
| Some of the PrEP clients we have spoken to in the clinic say they were advised to take PrEP by a family member, friend or someone else in the community. Can you tell me about;  What things you would consider when advising someone to take PrEP or not?  Who you would not advise to take PrEP?  Who you would advise to take PrEP? |
| How do you feel about PrEP in comparison to other HIV prevention strategies?  What are some things about PrEP that make you feel hopeful?  What are some things about PrEP that make you feel skeptical? |
| Can you tell me about any training or information you have received regarding PrEP?  Probe on whether it was informative  Probe on where and from whom they had training  Probe on would they like training / more training |
| 1. Where do you think PrEP should be delivered? |
| 1. Have you already been approached by someone who wanted to talk about PrEP? How did that conversation go?    1. Probe on what they discussed    2. Probe on whether they referred them to a clinic    3. Probe on why they discussed this with them |
| How do you think the conversation would go when trying to discuss PrEP with a member of your community?  Probe on where would the conversation take place  Probe on adolescents  Probe on men |
| If someone was uncomfortable discussing PrEP with you, where would you advise them to go? |
| 1. What can you do to inform your community better about the different HIV prevention methods available, including PrEP? |
| 1. Please tell us about whether you would support community engagement with PrEP.    1. Probe on promoting materials    2. Probe on helping with the risk assessments    3. Probe on referral to clinics |
| Thinking about the future, can you tell me about how PrEP will affect your community?  Probe on education  Probe on Children  Probe on Jobs  Probe on Illness |
| What do you think is the biggest challenge to preventing new HIV infections in Swaziland? |
| 1. Is there anything I haven’t asked you that I should have asked you? |

We have come to the conclusion of the topics I had prepared to discuss today. Are there any further comments you would like to add? **THANK YOU FOR YOUR TIME!**
