# Supplementary material for: “It’s hard for us men to go to the clinic. We naturally have a fear of hospitals.” Men’s risk perceptions, experiences and program preferences for PrEP: A mixed methods study in Eswatini
Source: PLoS One. 2020 Sep 23;15(9):e0237427. doi: 10.1371/journal.pone.0237427 (PMC7510987; doi:10.1371/journal.pone.0237427)
Supplement: S13 File — (DOCX) [file pone.0237427.s013.docx]

QUALITATIVE TOOL – COMMUNITY LEADER INTERVIEWS

**Community leaders in Swaziland**

As we went over in the consent, all of the information you provide will be kept confidential. Just as a reminder our interview will probably last around 45-60 minutes. Do you have any questions before we begin? May I start the recording? *[Start recording]* Njengoba sesivumelene, lonkhe lwati lotanginiketa lona lutawuba yimfihlo. Kukukhumbuta nje kutsi kukhulumisana kwetfu kutawutsatsa sikhatsi lesingaba ngu 45-60 wemaminishana. Engabe unawo yini umbuto ngaphambi kwekuba sicale? Ngingacala ngirekhode? [Cala ku rekhoda]

**Good [afternoon/morning] thank you for participating today**! I have asked you to meet with me in the hopes of learning more about your experience and perceptions related to a new HIV prevention strategy called pre-exposure prophylaxis (PrEP), You have been identified by men and women in your community as a key figure and community leader. We are interested to learn your thoughts regarding PrEP because you may have some influence regarding how PrEP is seen within your community. Some of the questions I will ask you may not want to answer and that is fine. Remember that your answers are confidential and participation is completely voluntary. Also please keep in mind that there are no right or wrong answers, I am interested in anything you can share with me. Siyabingelela [emini/ekuseni] futsi siyabonga kutsi ube yincenye yalokukhulumisana namuhla. Ngikucele kutsi sibe nekuhlangana ngelitsemba lekwati kabanti ngalohlangabetene nako kanye nemivo yakho mayelana neluhlelo lolusha lekuvikela ligciwane lengculaza, HIV, lekubitwa nge pre-exposure prophylaxis (PrEP). Siyakutsakasela kuva imivo yakho nge PrEP njengemuntfu loyinhloko lokheftwe bomake nabobabe nalongaba nesandla ekutseni bantfu bayibuka kanjani I PrEP emmangweni wakini. Leminye imibuto lengitakubuta yona ungafisa kungayiphendvuli ayikho inking lapho. Sicela ukhululeke ukhumbule kutsi kute imphendvulo lesingatsi ikahle nalesingatsi akusiyo mine ngitawujabulela lotangivetela kona kani nato timphendvulo takho tiyimfihlo.

Questions for PrEP community leaders Imibuto Ye PrEP yebaholi bemango:

| **Questions** |
| --- |
| 1. To begin, I was hoping you could tell me a bit more about yourself? Sesicala bengigatsandza ungitjele kabantana ngawe?    1. Can you tell me a bit more about your position and responsibilities? Ungangitjela kabanti ngesikhundla sakho kanye nalokwentako?    2. How do you represent your community? Uwumelelela kanjani umango wakho?    3. Please give me an example of a health matter you have discussed with a community member or the community generally. Ngisacela ungitjele ngesibonelo sendzaba yetempilo loke wayikhuluma nelilunga lemango noma umango wonkana. |
| 1. Can you talk me through how you have spoken with your community regarding HIV prevention strategies?   Ngisacela ungilandze nje kutsi uke wakhuluma kanjani nemango mayelana netinhlelo tekuvikela ligciwane lengculaza (HIV) |
| Please tell me about what makes advising your community regarding matters relating to HIV and sex difficult.  Ngicela ungitjele kutsi yini leyenta kululeka umango wakho mayelana netindzaba telicansi kanye ne HIV kube lukhuni  Probe on culture Butisisa nge masiko.  Probe on religion Butisisa ngetenkholo  Probe on gender Butisisa ngetebulili  Probe on age Butisisa ngeminyaka  Probe on knowledge levels Butisisa ngemazinga elwati  Probe on stigma Butisisa ngetekucwayana |
| Please tell me if you have received any training regarding HIV prevention?  Probe on who gave the training?  Probe on when the training was received?  Ask if they would be open to more training. |
| Can you tell me about any training or information you have received regarding PrEP?  Ngicela ungitjele ngelwati noma lucecesho tsite lolutfolile mayelana na PrEP  Probe on whether it was informative Butisisa kutsi lwaba lusito yini.  Probe on where and from whom they had training Butisisa kutsi lolucecesho walutfola kuphi futsi walunikwa ngubani?  Probe on would they like training / more training Butisisa kutsi bangakudzinga yini kuceceshwa lokwengetiwe |
| What have you heard about PrEP? Yini loke wayiva nge PrEP? |
| How do you feel about PrEP as an HIV prevention method? Utiva kanjani wena nge PrEP njengendlela yekuvikela ligciwane lengculaza (HIV) |
| Who do you think PrEP should be for? Ucabanga kutsi i PrEP ilungele bobani? |
| Who do you think PrEP shouldn’t be for? Ucabanga kutsi i PrEP ayilungeli bobani? |
| Can you tell me what you think PrEP means for your community? Ucabanga kutsi PrEP ishoni emangweni wakini? |
| Can you tell me about who you think will benefit the most from PrEP in your community?  Ngicela ungitjele kutsi bobani locabanga kutsi batawusitakala kakhulu emangweni wakini nge PrEP? |
| Some of the PrEP clients we have spoken to in the clinic say they were advised to take PrEP by a family member, friend or someone else in the community. Can you tell me about;  Labanye labasebentisa i PrEP lesike sakhulumisana nabo esibhedlela batsi baye balulekwe lilunga lemndeni, umngani noma lomunye nje emangweni kutsi bayitsatse. Ngicela ungitjele ngaloku;  What things you would consider when advising someone to take PrEP or not?  Yini tintfo longatibuka uma ululeka umuntfu ekutseni uyamutsatsa PrEP noma cha?  Who you would not advise to take PrEP? Ngubani longameluleka kutsi angamutsatsi PrEP?  Who you would advise to take PrEP? Ngubani longameluleka kutsi atsatse PrEP? |
| How do you feel about PrEP in comparison to other HIV prevention strategies?  Utiva kanjani ngaye PrEP uma ucatsanisa naletinye tindlela tekuvikela ligciwane lengculaza (HIV)  What are some things about PrEP that make you feel hopeful? Ngutiphi tintfo letikwenta utive unelitsemba nga PrEP?  What are some things about PrEP that make you feel skeptical? Ngutiphi tintfo letikwenta ube nekungabata nga PrEP? |
| 1. In the demonstration projects, less than 30% were men.    1. What is your explanation why men ddo not come forward in great numbers?    2. What do you think could be done to improve male uptake for PrEP?    3. What could be done in communities to motivate men to take up PrEP? |
| 1. Where do you think PrEP should be delivered? Ucabanga kutsi lusito lwaka PrEP lungatfolakala kuphi? 2. If you could design a new PrEP delivery model for men, what would this look like? |
| 1. Have you already been approached by someone who wanted to talk about PrEP? How did that conversation go? Uke waba khona yini umuntfu loke weta kuwe wafuna kukhuluma nga PrEP? Leyo nkulumo yahamba njani?    1. Probe on what they discussed Butisisa kutsi bakhuluma ngani    2. Probe on whether they referred them to a clinic Butisisa kutsi baye babatfumela yini esibhedlela    3. Probe on why they discussed this with them Butisisa kutsi kungani bakhuluma loku nabo |
| How do you think the conversation would go when trying to discuss PrEP with a member of your community?  Uma ungazama kukhulumisana nelilunga lemango nga PrEP ucabanga kutsi loko kukhulumisana kungahamba njani?  Probe on where would the conversation take place Butisisa kutsi loko kukhulumisana kungaba kuphi  Probe on adolescents Butisisa ngetintfombi nemajaha (labasha)  Probe on men Buta ngemadvodza |
| If someone was uncomfortable discussing PrEP with you, where would you advise them to go?  Uma umuntfu ativa angakakhululeki kukhulumisana nawe nga PrEP ungameluleka kutsi aye kuphi? |
| 1. What can you do to inform your community better about the different HIV prevention methods available, including PrEP?   Yini longayenta kwatisa umango wakho kancono ngetindlela letehlukahlukene letikhona tekuvikela ligciwane lengculaza (HIV) lokufaka ekhatsi i PrEP? |
| 1. Please tell us about whether you would support community engagement with PrEP.   Ngicela ungitjele kutsi ungaba yini nekusekela kutsi umango wakini ungenele i PrEP   - 1. Probe on promoting materials Butisisa ngetintfo tekukhulisa lwati   2. Probe on helping with the risk assessments Butisisa ngekusita ekuhloleni bungoti lobungaba khona   3. Probe on referral to clinics Butisisa ngekudlulisela bantfu etibhedlela |
| Thinking about the future, can you tell me about how PrEP will affect your community?  Uma ucabanga lutawuba njani lushinto emangweni wakho lolubangelwa yi PrEP uma sesicabangela likusasa.  Probe on education Butisisa ngetemfundvo  Probe on Children Buta ngebantfwana  Probe on Jobs Buta ngematfuba emisebenti  Probe on illness Butisisa ngekuphatseka noma kugula |
| What do you think is the biggest challenge to preventing new HIV infections in Swaziland?  Ucabanga kutsi yini tingcinamba letinkhulu eveni lonkhana ekuvimbeni kutselelana kweligciwane legculaza (HIV)? |
| 1. Is there anything I haven’t asked you that I should have asked you?   Kukhona yini lengingakakubuti lova ngatsi bekufanele sikhulumisane ngako? |

We have come to the conclusion of the topics I had prepared to discuss today. Are there any further comments you would like to add? **THANK YOU FOR YOUR TIME!**

Sesifike emaphetselweni alokukhulumisa kwetfu namuhla. Kukhona yini lokunye kuphawula longatsandza kukungeta? **NGIYABONGA KAKHULU SIKHATSI SAKHO!**
